# O-versus S-Metal Coordination of the Thiocarboxylate Group: An NMR Study of the Two Tautomeric Forms of the Ga(III)-Photoxenobactin E Complex

**DOI:** 10.1021/acs.inorgchem.3c04076

**Published:** 2024-02-22

**Authors:** Larissa Buedenbender, Lucía Ageitos, Marta A. Lages, Carlos Platas-Iglesias, Miguel Balado, Manuel L. Lemos, Jaime Rodríguez, Carlos Jiménez

**Affiliations:** † CICA − Centro Interdisciplinar de Química e Bioloxía e Departamento de Química, Facultade de Ciencias, Universidade da Coruña, 15071 A Coruña, Spain; ‡ Departamento de Microbiología y Parasitología, Instituto de Acuicultura, Universidade de Santiago de Compostela, 15782 Santiago de Compostela, Spain

## Abstract

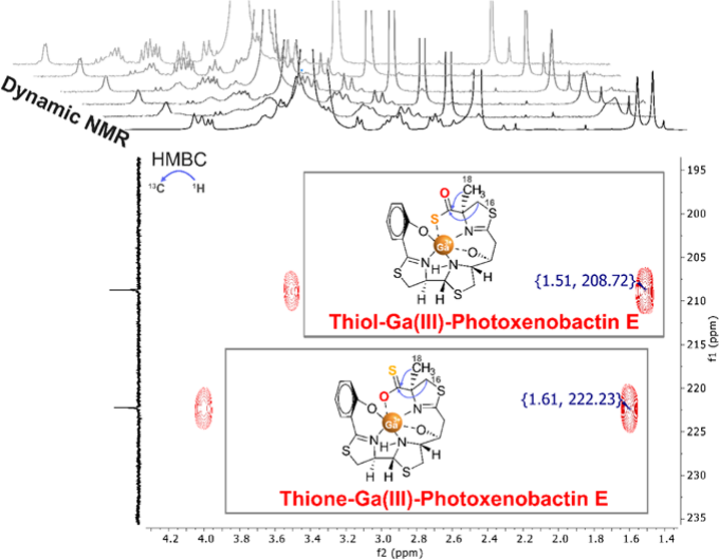

Photoxenobactin E (**1**) is a natural product with an unusual thiocarboxylic acid terminus recently isolated from an entomopathogenic bacterium. The biosynthetic gene cluster associated with photoxenobactin E, and other reported derivatives, is very similar to that of piscibactin, the siderophore responsible for the iron uptake among bacteria of the *Vibrionaceae* family, including potential human pathogens. Here, the reisolation of **1** from the fish pathogen *Vibrio anguillarum* RV22 cultured under iron deprivation, its ability to chelate Ga(III), and the full NMR spectroscopic characterization of the Ga(III)-photoxenobactin E complex are presented. Our results show that Ga(III)-photoxenobactin E in solution exists in a thiol–thione tautomeric equilibrium, where Ga(III) is coordinated through the sulfur (thiol form) or oxygen (thione form) atoms of the thiocarboxylate group. This report represents the first NMR study of the chemical exchange between the thiol and thione forms associated with thiocarboxylate-Ga(III) coordination, including the kinetics of the interconversion process associated with this tautomeric exchange. These findings show significant implications for ligand design as they illustrate the potential of the thiocarboxylate group as a versatile donor for hard metal ions such as Ga(III).

## Abstract 2

Ga(III)-photoxenobactin E in solution exists in a thiol–thione tautomeric equilibrium, where Ga(III) is coordinated through the S or O atoms of the thiocarboxylate group. This is the first NMR study describing the chemical exchange between the thiol and thione forms in thiocarboxylate-Ga(III) coordination and the associated kinetics of interconversion. The results suggest that this motif can be useful for the preparation of new ligand families.

## Introduction

The interest in metal complexes of thiocarboxylate ligands has increased in the last years not only from a structural point of view but also from synthetic aspects due to their interesting properties and their involvement in some biological processes.^1^ Because these ligands contain both “soft” sulfur and “hard” oxygen donor sites, they enable the incorporation of hard, soft, and borderline metal ions into a coordination compound, as well as the stabilization of unusual coordination numbers and geometries.^2^ Metal thiocarboxylate complexes are being used to prepare several types of sulfide materials that exhibit interesting optical, electronic, and conductive properties.^3−5^ They can be used to form polymeric compounds with electrical and luminescent features^6,7^ and to prepare new catalysts for organic synthesis^8^ such as the azide–alkyne cycloaddition reaction.^9^ Thiocarboxylate complexes have also shown to be effective in a variety of biological applications, such as enzymatic catalysis and drug delivery.^10^ Further applications of thiocarboxylate complexes include their use in a variety of environmental problems such as water purification and remediation of contaminated soils.^11,12^

**Scheme 1:**
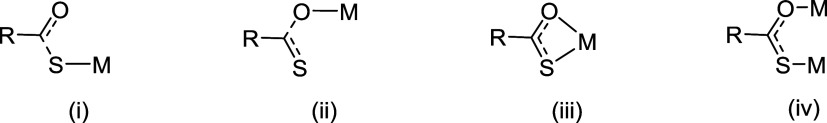
Thiocarboxylate Coordination Modes with Metal Ions as Monodentate Ligand through (i) the Sulfur Atom or (ii) the Oxygen Atom, (iii) as Chelate Bidentate (μ_1_-(O,S)) or (iv) Bridging Bidentate (μ_2_-(O,S)) Ligand (M = metal ion).

Although the tautomerism of thiocarboxylic acids as a mixture of thiol and thione forms has been studied on a number of occasions,^13−15^ the tautomerism of their corresponding thiocarboxylate complexes has received less attention. Four coordination modes have been suggested for thiocarboxylate complexes of metals, depending on the nature of the metal ion and the solvent (Scheme 1).^16,17^ The coordination behavior of the thiocarboxylate ligand toward the metal was studied by spectral analysis using infrared, electronic, and Mössbauer spectra. However, NMR studies of tautomerism between some of the mentioned coordination modes have not been reported so far.

There are a number of studies of the synthesis, structures, and applications of metal complexes bearing thiocarboxylate ligands;^1^ however, only a few examples of gallium–thiocarboxylate complexes have been reported so far. Nevertheless, the coordination chemistry of thiocarboxylate ligands remains underexplored, particularly in comparison with carboxylate analogues. The first dialkyl gallium thioacetate was published in 1971, but the first example of a structurally characterized gallium thiocarboxylate was methyl gallium thioacetate, Ga(SCOMe)_2_Me(3,5-dimethylpyridine) reported in 1996 as a novel single-source precursor for gallium thin films by aerosol-assisted chemical vapor decomposition (CVD).^18^ For the purpose of the present work, it is important to notice that the ^13^C NMR spectrum in bencene-*d*_6_ for this compound displays the carbonyl chemical shift at 201.95 ppm. Some years later, the synthesis and structural characterization of gallium thiobenzoates were reported. The treatment of thiobenzoic acid with one mol equivalent of trialkyl gallium, ^*t*^Bu_3_Ga or Me_3_Ga, yielded ^*t*^Bu_2_Ga[S(O)CPh] or Me_2_Ga[S(O)CPh], respectively. Their corresponding ^13^C NMR spectra in CDCl_3_ display the carbonyl chemical shifts at 221.98 and 218.81 ppm, respectively. In contrast, when Me_3_Ga is treated with three equivalents of thiobenzoic acid, a crystalline compound Ga[S(O)CPh]_3_ is obtained. Its crystal data, obtained by X-ray diffraction, showed a central hexacoordinate gallium atom bonded to three thiobenzoate moieties acting as bifunctional SO ligands. Its corresponding ^13^C NMR spectrum in CDCl_3_ showed a carbonyl chemical shift at 212.33 ppm.^19^

Natural thiocarboxylic acid-containing metabolites are extremely rare and so far, to the best of our knowledge, only nine natural products have been fully characterized due to the instability of these metabolites.^20−25^ Photoxenobactin E (**1a**) is such a natural product with an unusual thiocarboxylic acid terminus recently isolated by Shi and co-workers from an entomopathogenic bacterium.^25^ The *pxb* biosynthetic gene cluster (BGC) associated with photoxenobactin E (PxbE), and other reported derivatives, is very similar to the *irp* BGC encoding piscibactin (Pcb, **1b**), the siderophore responsible for iron uptake in the fish pathogenic Gram-negative bacterium *Photobacterium damselae* subsp. *piscicida*.^26^ The *irp* BGC is widespread among bacteria of the *Vibrionaceae* family including potential human pathogens^26−30^ and contains an additional heterocyclization–adenylation–thiolation (Cy_4_-A_2_-T_6_) module. This module was thought to be either nonfunctional or to be involved in the synthesis of a cryptic metabolite containing an additional thiazoline, but to date has not been identified.^26^ An identical module was described for the *pxb* BGC encoding for photoxenobactin E,^31^ suggesting that *Vibrionaceae* might also be able to produce photoxenobactin-like metabolites. Siderophores are low-molecular-weight metabolites that are produced by microorganisms to acquire essential iron directly from the environment.^32^ The ability to scavenge iron from iron-deficient environments determines the growth and survival of the microorganism, and siderophores represent important virulence factors for pathogenic bacteria.^33^ Pcb was characterized as its Ga(III) complex due to its high instability in its *apo*-form.^26^ Surprisingly, the metal binding ability of photoxenobactin E was not detected when its isolation was reported.^25^

Reinvestigation of the fish pathogen *Vibrio anguillarum* RV22, cultured under iron deprivation, allowed us to isolate the siderophore photoxenobactin E as its Ga(III) complex (**2**). The coordination chemistry of Ga(III) and Fe(III) displays many similarities: they have the same charge (both 3+), similar ionic radii (Fe(III) = 0.65 Å, Ga(III) = 0.62 Å for coordination number six),^34^ preferred coordination number of six, and they are hard Lewis acids.^35^ The diamagnetic character of Ga(III), in contrast to the paramagnetic character of Fe(III), enables NMR studies of the Ga(III) thiocarboxylate complexes.

**Figure 1:**
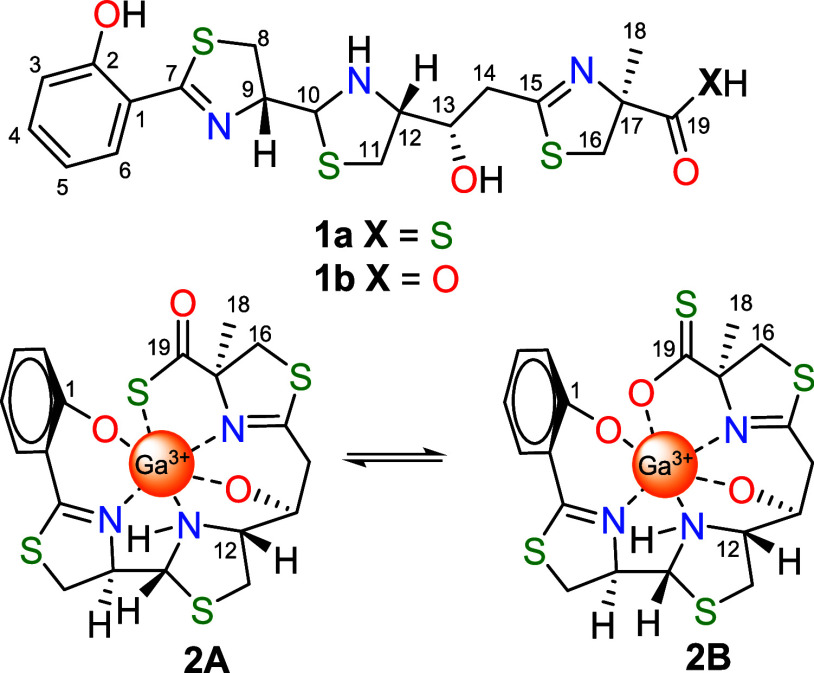
*Apo*-forms of photoxenobactin E (**1a**) and piscibactin (**1b**) as well as *holo*-thiol (**2A**) and *holo*-thione (**2B**) forms of photoxenobactin E as the Ga(III)-photoxenobactin E complex (**2**).

In this work, we report the reisolation of photoxenobactin E (**1a**) from the fish pathogen *V. anguillarum* RV22, its ability to chelate Ga(III), and the full NMR spectroscopic characterization of the Ga(III)-photoxenobactin E complex, via experimental and computational investigations. Our results showed that the Ga(III)-photoxenobactin E complex (**2**) exists as a tautomeric equilibrium mixture, where Ga(III) is bound either through the sulfur (**2A**) or through the oxygen (**2B**) of the thiocarboxylate (Figure 1). Moreover, we performed dynamic NMR to study the kinetic process of this tautomeric exchange. To the best of our knowledge, this is the first report in which the chemical exchange associated with thiocarboxylate-Ga(III) coordination of both the thiol and thione species was observed via NMR spectroscopy, allowing the analysis of kinetics of this tautomeric exchange.

## Results and Discussion

The isolation of photoxenobactin E (**1a**) by Bode and co-workers from entomopathogenic bacteria and the similarity of its biosynthetic gene cluster^25^ to that of piscibactin (**1b**),^26^ motivated us to investigate whether pathogenic *Vibrionaceae* are also able to biosynthesize this compound and to assess its metal-chelating properties.

Cell-free culture supernatants of strain *V. anguillarum* RV22 grown under iron-restricted conditions were chelated with Ga(acac)_3_. Formation of Ga(III) complexes stabilizes the siderophores and allows spectroscopic analysis via NMR due to the absence of paramagnetism in this metal ion. Solid Phase Extraction (SPE) using HLB cartridges of the cell-free culture supernatants followed by HPLC separation allowed the isolation of the Ga(III) complex of photoxenobactin E (**2**). The molecular formula of **2** was established based on the isotopic cluster distribution due to the presence of gallium and the HRESIMS [M + H]^+^ ion at *m*/*z* 535.9719 (calculated for C_19_H_21_N_3_O_3_S_4_^69^Ga *m*/*z* 535.9722 [M + H]^+^), which matched that of the Ga(III)-photoxenobactin E complex (Figure S1, Supporting Information).

### Characterization of the Ga(III)-Photoxenobactin E Complex by NMR and IR Spectroscopy

Comparison of 1D and 2D NMR spectral data of **2** in DMSO-*d*_6_ with those reported for *apo*-photoxenobactin E (**1a**)^25^ allowed the confirmation of its structure. The carbon and proton chemical shifts were very similar in both compounds (Table 1). However, proton chemical shifts at positions near the Ga(III) ion: the methyl C-18 and methylene C-16 protons of the methylthiazoline moiety, the proton bonded to the nitrogen (10-NH) in the thiazolidine ring, and the proton at position C-12, exhibited duplicate signals in the ^1^H NMR spectrum of **2**. More specifically, the chemical shift corresponding to CH_3_-18 resonated as two distinct methyl proton singlets at δ_H_ 1.51 and δ_H_ 1.61, which correlated in the HSQC experiment with carbons at δ_C_ 24.56 and δ_C_ 25.69, respectively (Figure S6). The ^1^H NMR methylene signals at C-16 were observed at δ_H_ 3.16/3.51 and δ_H_ 3.34/4.01, which correlated in the HSQC experiment with the carbon chemical shifts at δ_C_ 38.17 and δ_C_ 42.41, respectively. Two carbon resonances at higher chemical shifts were observed at δ_C_ 208.72 and δ_C_ 222.23 which correlated in the HMBC experiment to the CH_3_-18 protons at δ_H_ 1.51 and δ_H_ 1.61, respectively (Figure S7). Two proton chemical shift signals were observed for 10-NH of the thiazolidine ring at δ_H_ 6.37 and δ_H_ 6.64. Finally, the chemical shift of the methine proton at C-12 was observed as two multiplet signals at δ_H_ 3.70 and δ_H_ 3.62, which correlated in the HSQC experiment with carbons at δ_C_ 68.40 and δ_C_ 68.74, respectively. The carbon chemical shift of δ_C_ 208.72 agrees with the C=O resonance of the thiol form of the thiocarboxylate group as reported by Bode and co-workers,^25^ while the carbon chemical shift at δ_C_ 222.23 likely corresponds to the C=S resonance of the thione form of the thiocarboxylate group.^36^

**Table 1:** ^1^H and ^13^C NMR Data of *apo*-Photoxenobactin E (**1**, Reported by Bode and Co-Workers^25^) and for Ga(III)-Photoxenobactin E Tautomers (**2A/B**) at 298.15 K (Slow Exchange) and at 348.15 K (**2T**) (Fast Exchange) in DMSO-*d*_6_^a^

***Apo*****-photoxenobactin E**			**Ga(III)-photoxenobactin E complex**					
	**1a**		**2A** (thiol-form)		**2B** (thione-form)		**2T**	
	298 K (800/200 MHz)^25^		298 K (500/125 MHz)		298 K (500/125 MHz)		348 K (400/100 MHz)^b^	
**#**	δ_H_ (mult., *J* in Hz)	δ_C_, mult.	δ_H_ (mult., *J* in Hz)	δ_C_, mult.	δ_H_ (mult., *J* in Hz)	δ_C_, mult.	δ_H_ (mult., *J* in Hz)	δ_C_, mult.
**1**		172.4, C		166.20, C		166.25, C		n.d.
**2**	6.50 (d, 8.5)	124.4, CH	6.67 (m)	123.25, CH	6.67 (m)	122.68, CH	6.68 (d, 8.8)	122.2, CH
**3**	7.09 (td, 8.5, 1.8)	133.9, CH	7.32 (m)	135.39, CH	7.33 (m)	135.67, CH	7.30 (m)	134.6, CH
**4**	6.32 (br t, 7.3)	112.1, CH	6.63 (m)	115.27, CH	6.63 (m)	114.73, CH	6.64 (t, 7.5)	114.6, CH
**5**	7.16 (dd, 7.9, 1.8)	132.3, CH	7.27 (m)	131.67, CH	7.27 (m)	131.71, CH	7.29 (d, 7.5)	130.7, CH
**6**		116.7, C		115.39, C		115.85, C		n.d.
**7**		172.1, C		174.83, C		176.41, C		n.d.
**8**	3.09 (ov)	33.7, CH_2_	3.34 (ov)	33.36, CH_2_	3.34 (ov)	33.05, CH_2_	3.36 (dd, 12.3, 11.3)	32.6, CH_2_
	3.45 (ov)		3.54 (ov)		3.54 (ov)		3.57 (dd, 11.3, 8.6)	
**9**	4.40 (ddd, 13.1, 10.3, 7.5)	78.7, CH	4.62 (m)	75.67, CH	4.61 (m)	75.72, CH	4.59 (ddd, 12.3, 9.9, 8.6)	75.3, CH
**10**	4.71 (dd, 10.3, 6.8)	70.6, CH	4.88 (dd, 9.9, 6.6)	68.99, CH	4.88 (dd, 9.9, 6.6)	68.85, CH	4.83 (dd, 9.9, 6.6)	68.6, CH
**10-NH**	5.31 (dd, 10.3, 6.9)		6.37 (dd, 9.5, 6.7)*		6.64*		6.30 (br t)	
**11**	3.45 (dd, 12.3, 7.6)	37.5, CH_2_	2.99 (m)	37.76, CH_2_	2.99 (m)	37.23, CH_2_	3.01 (ov)	37.0, CH_2_
	3.03 (br t, 11.4)		3.50 (m)		3.50 (m)		3.52 (dd, 12.4, 7.3)	
**12**	**3.72** (dd, 18.1, 10.3)	66.3, CH	**3.70** (m)**	68.40, CH	**3.62** (m)**	68.74, CH	**3.73** (m)	68.1, CH
**13**	3.99 (br s)	67.8, CH	4.08 (m)	70.07, CH	4.08 (m)	70.16, CH	4.14 (m)	69.8, CH
**13-OH**	7.52 (s)							
**14**	2.88	40.0, CH_2_	2.73 (brt, 14.4)	40.31, CH_2_	2.73 (br t, 14.4)	40.83, CH_2_	2.75 (d, 17.3)	40.3, CH_2_
	3.08		2.92 (m)		2.92 (m)		2.95 (dd, 17.3, 5.1)	
**15**		170.6, C	-	180.21, C		181.92, C		n.d.
**16**	**3.59** (d, 11.5)	39.4, CH_2_	**3.16 (**d, 11.5)	38.17, CH_2_	**3.34** (ov)	42.41, CH_2_	**3.25** (d, 11.2)	39.2, CH_2_
	**3.15** (d, 11.5)		**3.51** (ov)		**4.01** (d, 11.1)		**3.79** (ov)	
**17**		93.1, C		88.80, C		88.64, C		n.d.
**18**	**1.56 (s)**	25.6, CH_3_	**1.51 (s)**	24.56, CH_3_	**1.61 (s)**	25.69, CH_3_	**1.60 (s)**	24.7, CH_3_
**19**		**212.1**, C		**208.72**, C		**222.23**, C		n.d.

a ov = overlapped resonances; n.d. = not detected; *,** = interchangeable signals.

b assigned by ^1^H,^1^H–COSY and ^1^H,^13^C-HSQC NMR experiments, hence quaternary carbons are not listed.

Closer inspection of the ^13^C NMR spectrum of **2**, showed duplication of all carbon chemical shifts (Table 1 and Figure S4). Most of them displayed minor chemical shift differences that ranged between 0.1 to 1.5 ppm (Figure S4), but large differences were observed for carbons at C-16 (Δδ 4.24 ppm) and C-19 (Δδ 13.51 ppm) positions.

This behavior is characteristic of the presence of two complex species, where the variation in chemical shift is due to the different environments of the corresponding nuclei. The NOESY spectrum of **2** showed evidence of chemical exchange through cross-peaks between the two sets of 10-NH-signals of the thiazolidine ring (Figure S8). All of these data suggest that Ga(III)-photoxenobactin E complex (**2**) occurs as a tautomeric mixture of its thiol-**2A** and thione-**2B** forms, where the Ga(III) ion is coordinated to the sulfur or the oxygen, respectively (Figure 1). Integration of the CH_3_-18 resonances in the ^1^H NMR spectrum of **2** in DMSO-*d*_6_ indicated that the two tautomer species occurred in a ∼51:49 ratio at 298 K (Figure S3), with the thiol population being slightly higher compared to that of the thione form. Based on this ratio, we could differentiate the carbon chemical shifts of each tautomeric form in the ^13^C NMR spectrum of **2** (Table 1 and Figure S4).

Over time, we observed an increase of a singlet signal at 1.49 ppm in the ^1^H NMR spectra of **2** that was assigned to the methyl C-18 resonance of the Ga(III)-piscibactin complex. The presence of this compound was confirmed by the HRESIMS-(+) of an aliquot of the NMR tube, which shows the [M + H]^+^ ion at *m*/*z* 519.9948 corresponding to Ga(III)-piscibactin complex (**2**). We suggest that the Ga(III)-piscibactin complex must be the hydrolysis product of the Ga(III)-photoxenobactin E complex (**2**) since the hydrolysis of thiocarboxylic acids to carboxylic acids has been reported previously. Cortese et al. observed the hydrolysis of pyridine-2,6-bis(thiocarboxylic acid) in aqueous solutions through the stepwise release of H_2_S, particularly when the compound was chelated with Bi(III), Cr(III), and Pb(II).^11^ Recently, the transformation of thiocarboxylic acids into carboxylic acids via a visible-light-promoted atomic substitution was described with DMSO as the oxygen source.^37^

The absorption bands observed in the IR spectrum of **2** at 1606 cm^–1^ (very sharp and strong) and 982 cm^–1^ (weak) (Figure S2) were assigned to the ν(C=O) and ν(C=S) stretching vibration bands, respectively. The increase of both ν(C=O) and ν(C=S) in relation to those of sodium salts of monothiobenzoic acid (ν(C=O) at 1500 and ν(C=S) at 960 cm^–1^), used as a reference, confirmed coordination to gallium through the sulfur and oxygen atoms of the thiocarboxylate group.^16,17^

### DFT Calculations of NMR Chemical Shifts of Tautomers 2A and 2B

The thiol-**2A** and thione-**2B** forms were optimized by using DFT at the B3LYP/6-31+G(d,p) level. Attempts were performed to compute different octahedral geometries involving a bidentate coordination of the thiocarboxylate group to Ga(III) through both S and O atoms, instead of the lone pair of the N atom of the thiazoline ring. However, we found that the thiocarboxylate group always coordinates to Ga(III) as either a thiol or a thione form, as shown in Figure 1 for **2A** and **2B**, respectively. Their ^13^C NMR chemical shifts were theoretically determined at the mPW1PW91/6-311+G(2d,p) level and then corrected by the computed Tantillo’s slope and intercept of −1.0490 and 186.6525, respectively.^38^ The ^13^C NMR chemical shifts obtained by this methodology were 210.3 ppm for the thiol- and 225.6 ppm for the thione forms, which were in very good agreement with the experimental carbon chemical shifts of **2** in DMSO-*d*_6_ at 208.72 and 222.23 ppm, respectively.

### Dynamic NMR Studies

**Figure 2:**
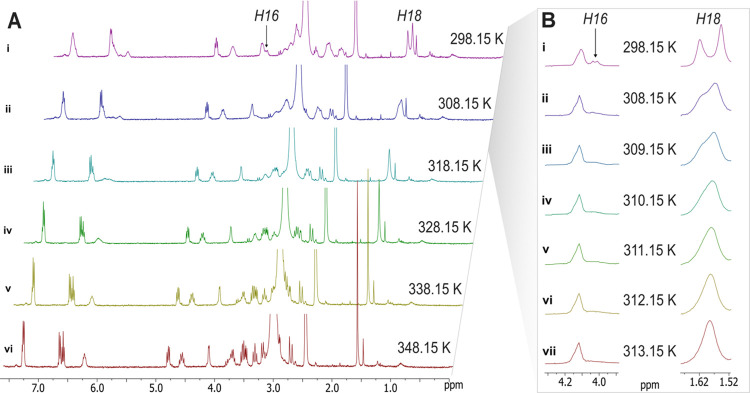
(A) Dynamic ^1^H NMR of the tautomeric mixture of the Ga(III)-photoxenobactin E complex (**2**) measured in DMSO-*d*_6_ at variable temperatures: 298.15 to 348.15 K (400 MHz). (B) Expansion of regions from 1.5 to 1.7 ppm, showing the proton chemical shift corresponding to the methyl H-18 signal, and from 3.9 to 4.3 ppm, showing the proton chemical shift at δ_H_ 4.01 ppm corresponding to one of the methylene protons at C-16 (DMSO-*d*_6_, 400 MHz, 298.15–313.15 K).

To confirm the existence of a tautomeric equilibrium between species **2A** and **2B**, we performed NMR experiments at variable temperatures (Figure 2). When the temperature was increased, the proton chemical shifts in the ^1^H NMR spectrum of the Ga(III)-photoxenobactin E complex (**2**) recorded in DMSO-*d*_6_ (400 MHz) exhibited a better resolution and the signal duplication disappeared (Figure 2, spectrum vi). For instance, slow exchange was observed at room temperature (298.15 K) for the signals of the C-18 methyl group for each tautomeric form as two sharp singlets separated by 25.33 Hz (Figure 2B, spectrum i). When the sample was warmed (303–313 K), the two singlet signals broadened, coalesced, and then started to sharpen and overlap (Figure 2B, spectra iv–vii). Fast exchange occurs at temperatures above 313.15 K and the C-18 methyl group is displayed as a single averaged peak in the ^1^H NMR spectrum.

The coalescence effect was also observed for the methylene protons at position C-16. At room temperature (298.15 K), the diastereotopic methylene protons in **2A** and **2B** resonated as two chemical shift pairs at δ_H_ 3.16/3.51 and δ_H_ 3.34/4.01 (Figure 2B, spectrum i), respectively. Interestingly, as we increased the temperature in the NMR experiments, the proton at δ_H_ 4.01 became broader and then disappeared at 313.15 K (Figure 2Bvii), indicating the presence of a dynamic process.

On the other hand, at higher temperatures, a new signal (broad doublet) appeared at δ_H_ 3.23 (Figure S10A), showing a similar coupling constant (11.2 Hz) as the one observed at δ_H_ 3.16 at 298.15 K. At 348.15 K, the selective irradiation of the signal at δ_H_ 3.23 in a 1D-TOCSY experiment revealed its diastereotopic partner at δ_H_ 3.79 (Figure S10B).

The assignment of the two methylene protons at δ_H_ 3.23 and 3.79 at C-16 (DMSO-*d*_6_, 400 MHz, 348.15 K, Figure S10B) was confirmed by the correlation between those protons observed in ^1^H–^1^H COSY spectrum of **2** (DMSO-*d*_6_, 400 MHz, 348.15 K, Figure S10C). These chemical shifts are the averaged values for both tautomeric forms **2A** and **2B** in a fast-interchanging regime of the two pairs of diastereotopic protons seen at 298.15 K. The ^1^H–^13^C-HSQC NMR spectrum correlation between δ_H_ 3.23 and δ_C_ 39.5 (Figures S10D) corroborated the assignment of C-16 as well as the rest of the NMR resonances as weight-averaged values of the two tautomeric forms (**2T**) in the fast chemical exchange process (Table 1).

**Figure 3:**
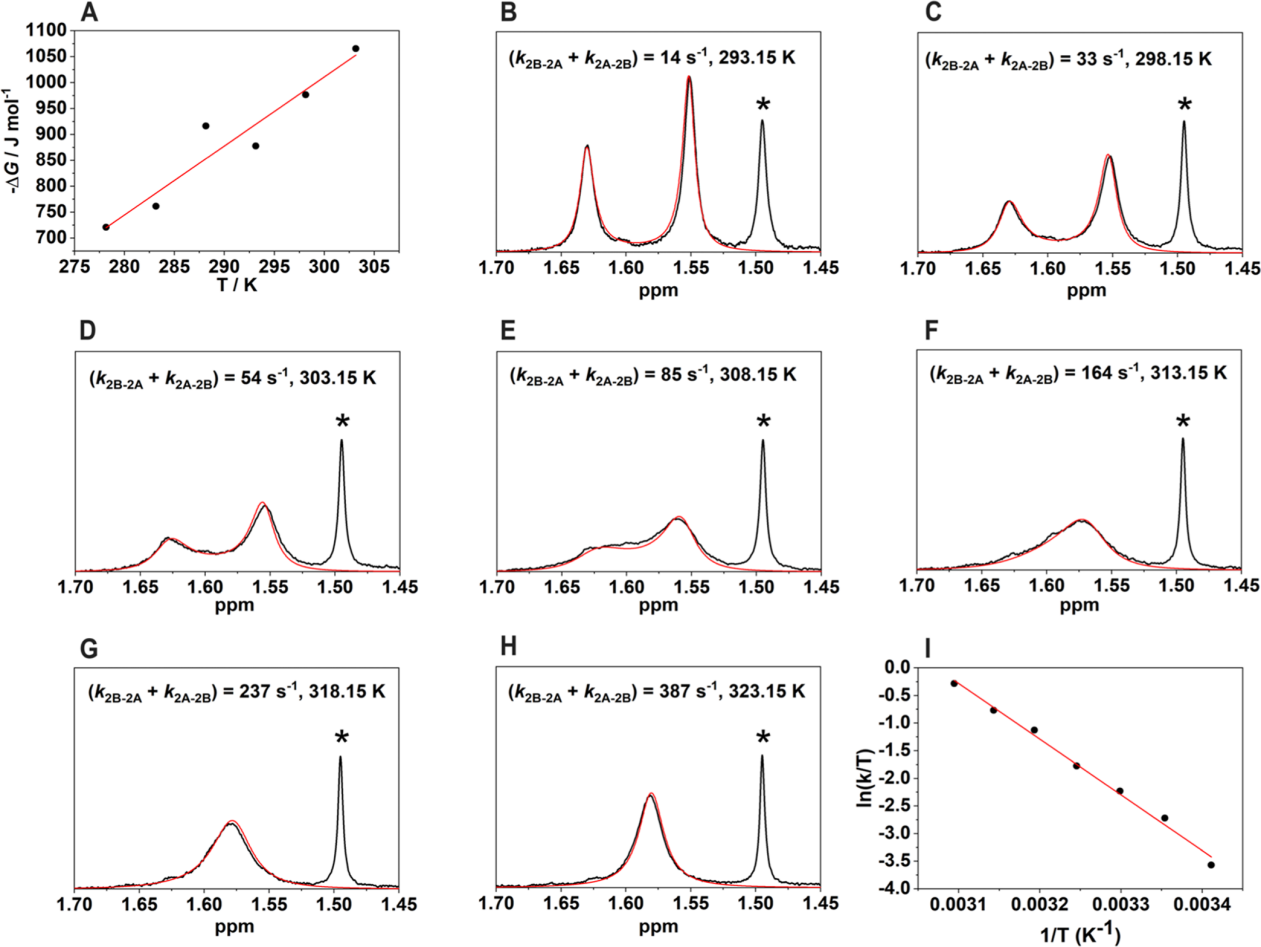
(A) Free energy values versus temperature for the **2B** ⇆ **2A** tautomeric equilibrium obtained from the integration of C-18 signals in DMSO-*d*_6_:CD_3_OD (9:1) solution; (B–H) Experimental (black) and simulated (red) ^1^H NMR spectra and rate constants obtained at different temperatures; (I) Eyring plot for the **2B** → **2A** interconversion. (*) The signal at 1.49 ppm corresponds to the C-18 signal of Ga(III)-piscibactin as the hydrolysis product of Ga(III)-photoxenobactin E.

Due to the high freezing point of DMSO-*d*_6_, we acquired the NMR experiments of Ga(III)-photoxenobactin E complex (**2**) at lower temperatures in a 9:1 deuterated solvent mixture of DMSO-*d*_6_:CD_3_OD (Figure S11). The separation of the two sharp signals (30.80 Hz) corresponding to the C-18 methyl groups of **2A** and **2B** observed in this solvent mixture at δ_H_ 1.55 and 1.63, respectively, was higher than that observed in DMSO-*d*_6_ (25.33 Hz). These resonances were subsequently used to characterize the tautomeric equilibrium defined as **2B** ⇆ **2A**. The relative intensities of the C-18 signals were obtained by deconvolution of the ^1^H NMR spectra in the temperature range 278.15–308.15 K, as above this temperature coalescence takes place. The relative populations of the two tautomers afforded the equilibrium constant at each temperature, which increases slightly from *K*_e_ = 1.37 at 273.15 K to 1.53 at 303.15 K, indicating that the relative population of **2A** progressively increases with temperature. A plot of Δ*G*^**2B**→**2A**^ = −RT ln *K*_e_ versus temperature gives a straight line (Figure 3A), with the corresponding linear fit yielding the reaction enthalpy (Δ*H*^**2B**→**2A**^ = +2980 ± 570 J mol^–1^) and reaction entropy (Δ*S*^**2B**→**2A**^ = +13.3 ± 2.0 J mol^–1^ K^–1^) for the tautomeric equilibrium. These data indicate that the higher population of the thiol form **2A** around room temperature is the result of a favorable entropy contribution, which compensates for the unfavorable enthalpy term. Our DFT calculations, using DMSO as the solvent, display a very small free energy difference between the two local minima, favoring the thiol form by only 0.52 kJ mol^–1^. This is in agreement with the experimental NMR data.

### Kinetics of Tautomeric Exchange

The kinetics of chemical exchange involving the tautomerization process were investigated by simulating the ^1^H NMR spectra at varying temperatures with the freeware WinDNMR (Figure 3B–H).^39^ The line widths of the C-18 methyl groups were simulated assuming that they are the result of an exchange process involving two sites with different populations. The analysis was performed using a rather broad temperature range of 293.15 to 323.15 K, incorporating spectra below and above coalescence, which occurs at ∼313 K. The populations of the two forms were obtained from the reaction enthalpy (Δ*H*^**2B**→**2A**^) and reaction entropy (Δ*S*^**2B**→**2A**^) values described above. The experimental spectra could be satisfactorily simulated with the rate constants (*k*^**2B**→**2A**^ + *k*^**2A**→**2B**^) shown in Figure 3. The rate constants for the **2B** → **2A** interconversion process (*k*^**2B**→**2A**^) were subsequently obtained by multiplying (*k*^**2B**→**2A**^ + *k*^**2A**→**2B**^) by the molar fraction of **2A**.^40^ The values of *k*^**2B**→**2A**^ obtained at different temperatures were analyzed using an Eyring plot according to eq 1, where *k*_b_ and *h* are Boltzmann and Planck constants, respectively, *R* is the gas constant, and Δ*H*^‡^ and Δ*S*^‡^ are the activation enthalpy and activation entropy.^41^
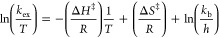
1

The Eyring plot affords a rather large Δ*H*^‡^ value of 83.9 ± 3.2 kJ mol^–1^, which is mainly associated with the energy cost required to break the Ga(III)-O bond in the **2B** thione form and reach the transition state. Similar Δ*H*^‡^ values were reported for the rotation of carboxylate groups in complexes with the trivalent lanthanide ions.^42,43^ The large enthalpy cost is compensated for in part by a positive Δ*S*^‡^ value (+60.0 ± 2.4 J mol^–1^ K^–1^). The sign of Δ*S*^‡^ is in agreement with the sign of the reaction entropy for the **2B** ⇆ **2A** equilibrium, as described above. This entropy change likely arises from the gain in degrees of freedom of the solvent molecules upon exposure of the less electronegative S atom to the solvent. This hypothesis is supported by the fact that the rotation of carboxylate groups in lanthanide complexes proceeds with a negligible activation entropy.^42^

### Solvent Effects

Since solvents are known to influence tautomerism, we proceeded to study this effect on the thione–thiol exchange process by running the NMR spectra in different deuterated solvents (Tables 1 and 2; Figures S12–S19).^44^ Although the best solubility was obtained in DMSO-*d*_6_ and in a DMSO-*d*_6_:CD_3_OD (9:1) mixture, the Ga(III)-photoxenobactin E complex (**2**) was also soluble in CDCl_3_ and partially soluble in THF-*d*_8_, but it was insoluble in CD_3_OD and D_2_O.

**Table 2:** ^1^H and ^13^C NMR Data Assignments of the Ga(III)-Photoxenobactin E Tautomers (**2A/B**) in DMSO-*d*_6_, CDCl_3_, and THF-*d*_8_ (500/125 MHz, 298.15 K)^a^

	**2A** (thiol-form)		**2B** (thione-form)		**2A** (thiol-form)		**2B** (thione-form)		**2A** (thiol-form)		**2B** (thione-form)	
	DMSO-*d*_6_		DMSO-*d*_6_		CDCl_3_		CDCl_3_		THF-*d*_8_		THF-*d*_8_	
*#*-mult	δ_H_ (mult., *J*)	δ_C_	δ_H_ (mult., *J*)	δ_C_	δ_H_ (mult., *J*)	δ_C_	δ_H_ (mult., *J*)	δ_C_	δ_H_ (mult., *J*)	δ_C_	δ_H_ (mult., *J*)	δ_C_
**1-C**		166.20		166.25		166.7		166.7		167.4		167.4
**2-CH**	6.67 (m)	123.25	6.67 (m)	122.68	6.86 (d, 8.7)	124.3	6.86 (d, 8.7)	124.3	6.75 (m)	124.2	6.75 (m)	124.2
**3-CH**	7.32 (m)	135.39	7.33 (m)	135.67	7.32 (ddd, 8.7, 7.2, 1.7)	137.0	7.32 (ddd, 8.7, 7.2, 1.7)	137.0	7.22 (t, 7.8)	135.8	7.22 (t, 7.8)	135.8
**4-CH**	6.63 (m)	115.27	6.63 (m)	114.73	6.66 (dd, 8.0, 7.2)	116.9	6.66 (dd, 8.0, 7.2)	116.9	6.55 (m)	116.0	6.55 (m)	116.0
**5-CH**	7.27 (m)	131.67	7.27 (m)	131.71	7.29 (dd, 8.0, 1.7)	132.1	7.29 (dd, 8.0, 1.7)	132.1	7.27 (m)	132.0	7.27 (m)	132.0
**6-C**		115.39		115.85		115.1		115.1		116.1		116.1
**7-C**		174.83		176.41		177.9		177.9		177.3		177.3
**8-CH**_**2**_	3.34 (ov)	33.36	3.34 (ov)	33.05	3.19 (br m)	34.5	3.19 (br m)	34.5	3.25 (ov)	34.4	3.25 (ov)	34.4
	3.54 (ov)		3.54 (ov)		3.53 (m)		3.53 (m)		3.52 (ov)		3.52 (ov)	
**9-CH**	4.62 (m)	75.67	4.61 (m)	75.72	4.72	75.9	4.72	75.9	4.64 (br m)	76.7	4.64 (br m)	76.7
**10-CH**_**2**_	4.88 (dd, 9.9, 6.6)	68.99	4.88 (dd, 9.9, 6.6)	68.85	4.74 (br m)	70.6	4.74 (br m)	70.6	4.75 (br m)	70.7	4.75 (br m)	70.7
**11-CH**_**2**_	2.99 (m)	37.76	2.99 (m)	37.23	3.16 (br m), 3.42 (br m)	39.4	3.16	39.4	3.13 (ov), 3.42 (ov)	38.6	3.13 (ov), 3.42 (ov)	38.6
	3.50 (m)		3.50 (m)				3.42 (br m)					
**12-CH**	3.70 (m)**	68.40	3.62 (m)**	68.74	3.83 (br m)	69.0	3.83 (br m)	69.0	3.81(br m)*	69.0**	3.72 (br m)*	69.3**
**13-CH**	4.08 (m)	70.07	4.08 (m)	70.16	4.34 (d, 3.8)	71.8	4.34 (d, 3.8)	71.8	4.28 (br m)	71.1	4.28 (br m)	71.1
**14-CH**_**2**_	2.73 (brt, 14.4)	40.31	2.73 (br t, 14.4)	40.83	2.67 (d, 17.0), 3.15 (br m)	42.1	2.67 (d, 17.0)	42.1	2.71 (m)	40.9	2.71 (m)	40.9
	2.92 (m)		2.92 (m)				3.15 (br m)		3.04 (ov)		3.04 (ov)	
**15-C**		180.21		181.92		180.2		180.2		182.2		182.2
**16-CH**_**2**_	3.16 (d, 11.5)	38.17	3.34 (ov)	42.41	n.d.	37.8	n.d.	43.4	3.16 (ov)	38.9	3.37 (ov), 4.08 (d, 11.6)	43.5
	3.51 (ov)		4.01 (d, 11.1)						3.50 (br m)			
**17-C**		88.80		88.64		90.7		90.7		90.8		90.8
**18-CH**_**3**_	1.51 (s)	24.56	1.61 (s)	25.69	1.84 (s)	27.7	1.84 (s)	27.7	1.68 (s)	25.8	1.76 (s)	26.6
**19-C**		208.72		222.23		210.3		224.4		208.7		222.7

a ov = overlapped resonances; n.d. = not detected; *^,^** = interchangeable signals.

The NMR spectra of **2** in CDCl_3_ (Figures S12–S15) showed that the proton and carbon chemical shift of the CH_3_-18 group coalesce at room temperature at δ_H_ 1.84/δ_C_ 27.7. The presence of the two tautomeric forms in CDCl_3_ was suggested by the two C-19 carbon resonances at δ_C_ 210.3 and δ_C_ 224.4 observed in the HMBC experiment of **2** (Figure S15). This was confirmed by the HMBC correlations between the CH_3_-18 protons at δ_H_ 1.84 and both C-19 carbon resonances at δ_C_ 210.3 and δ_C_ 224.4. Relative intensities of the HMBC cross-peaks from the CH_3_-18 protons to the thiol and thione C-19 carbons suggested that the thione-form (**2B**) was predominant (Figure S15). CDCl_3_ is less polar than DMSO-*d*_6_, and thus the ligation through the oxygen atom appears to be favored in nonpolar solvents.

1D and 2D NMR experiments of **2** recorded in THF-*d*_8_ (Figures S16–S19) showed duplicate signals, indicating the presence of the two tautomeric forms in this solvent. For example, two carbon resonances for C-19 at δ_C_ 208.7 and δ_C_ 222.7, were observed in the HMBC spectrum of **2** that correlated to CH_3_-18 protons at δ_H_ 1.68 and at δ_H_ 1.76, respectively (Figure S19). Furthermore, the CH_2_–16 protons were observed in this solvent as two chemical shift pairs at δ_C_ 38.9/δ_H_ 3.16 and 3.50 and at δ_C_ 43.5/δ_H_ 3.37 and 4.08 (Table 2). We could not determine the tautomer ratio of the thiol- and thione forms in this case because the CH_3_-18 methyl chemical shifts were under the residual protonated species of the deuterated solvent. However, intensities of the cross-peaks between CH_3_-18 protons and the two carbon resonances for C-19 in the HMBC spectrum (Figure S19) suggest that the thiol-form (**2A**) in THF-*d*_8_ is slightly more abundant in this solvent than in CDCl_3_.

Thus, experiments in different solvents indicate that the thiol form is more favored in polar solvents. This is reasonable, as coordination through the sulfur atom leaves the more electronegative O atom of the thiocarboxylate group exposed to the solvent, and thus, most likely, the thiol form is more efficiently solvated by polar solvents compared with the thione tautomer.

## Conclusions

In summary, in this study, we described for the first time the structure of the Ga(III)-photoxenobactin E complex (**2**) and its tautomeric equilibrium via NMR analysis where the Ga(III) can be coordinated through either the sulfur or the oxygen of the thiocarboxylate terminal. The thiol-form of Ga(III)-photoxenobactin E results in δ_C_ 208.7 (298.15 K, DMSO-*d*_6_, 500 MHz) while the thione form results in δ_C_ 222.2 (298.15 K, DMSO-*d*_6_, 500 MHz). These chemical shift assignments were supported by DFT calculations. ^1^H NMR line shape analysis of the signal at position C-18 of the Ga(III)-photoxenobactin E complex (**2**) estimated the temperature of coalescence in DMSO-*d*_6_ at 313.15 K. Using WinDNMR software and the Eyring equation, the kinetic parameters for the PxbE-Ga(III) tautomer were calculated. To the best of our knowledge, carbon NMR chemical resonances associated with the Ga(III)-bound thione-form have not been described in the literature. Bode and co-workers^25^ only observed thiol-associated NMR resonances (δ_C_ 212.1 in DMSO-*d*_6_) in the thiocarboxylate terminal of *apo*-photoxenobactin E.

The present work has important implications for ligand design, as we have demonstrated that the thiocarboxylate group can act as an efficient donor for hard metal ions, such as Ga(III), paving the way for the preparation of new ligand families containing this motif. Our results indicate that both O- and S-coordination can take place, with the interconversion among the two forms taking place in the ms time scale.

## Supporting Information Available

HRMS, IR, and NMR spectra of **2**; Cartesian coordinates obtained with DFT calculations (PDF)
