# Supplementary material for: O-versus S-Metal Coordination of the Thiocarboxylate Group: An NMR Study of the Two Tautomeric Forms of the Ga(III)-Photoxenobactin E Complex
Source: Inorg Chem. 2024 Feb 22;63(9):4176–84. doi: 10.1021/acs.inorgchem.3c04076 (PMC10915793; doi:10.1021/acs.inorgchem.3c04076)
Supplement: Supplementary file 1 — ic3c04076_si_001.pdf [file ic3c04076_si_001.pdf]

Supporting Information

O-*versus* S-Metal Coordination of the Thiocarboxylate Group: An NMR Study of the two Tautomeric Forms of the Ga(III)-Photoxenobactin E Complex

Larissa Buedenbender†, Lucía Ageitos†, Marta A. Lages‡, Carlos Platas-Iglesias†, Miguel Balado‡, Manuel L. Lemos‡, Jaime Rodríguez†*and Carlos Jiménez†*

† CICA – Centro Interdisciplinar de Química e Bioloxía e Departamento de Química, Facultade de Ciencias, Universidade da Coruña, 15071 A Coruña, Spain

‡ Departamento de Microbiología y Parasitología, Instituto de Acuicultura, Universidade de Santiago de Compostela, 15782 Santiago de Compostela, Spain

Corresponding Authors

* Jaime Rodríguez - CICA – Centro Interdisciplinar de Química e Bioloxía e Departamento de Química, Facultade de Ciencias, Universidade da Coruña, 15071 A Coruña, Spain; Email: jaime.rodriguez@udc.es

* Carlos Jiménez - CICA – Centro Interdisciplinar de Química e Bioloxía e Departamento de Química, Facultade de Ciencias, Universidade da Coruña, 15071 A Coruña, Spain; Email: carlos.jimenez@udc.es

Table of Contents

General experimental procedures. *S4*

Bacterial cultivation and siderophore isolation *S4*

NMR analysis. *S5*

DFT calculations. *S5*

Figure S1. HRESIMS spectrum of Ga(III)-photoxenobactin E (2) isolated from *V. anguillarum* RV22. .... S6

Figure S2. IR spectrum of Ga(III)-photoxenobactin E tautomers (**2A/B**) in EtOAc (%Transmittance) S6

A – NMR experiments obtained in DMSO-d_6. .... *S7*

Figure S3. ^{1}H NMR spectrum of the Ga(III)-photoxenobactin E tautomers (**2A/B**) (DMSO-d_6, 500 MHz, 298 K). *, ** = interchangeable signals S7

Figure S4. ^{13}C NMR spectrum of the Ga(III)-photoxenobactin E tautomers (**2A/B**) (DMSO-d_6, 125 MHz, 298 K). S8

Figure S5. ^{1}H,^{1}H-COSY NMR spectrum of the Ga(III)-photoxenobactin E tautomers (**2A/B**) (DMSO-d_6, 500 MHz, 298 K). S9

Figure S6. ^{1}H,^{13}C-HSQC NMR spectrum of the Ga(III)-photoxenobactin E tautomers (**2A/B**) (DMSO-d_6, 500 MHz, 298 K) S9

Figure S7. ^{1}H,^{13}C-HMBC NMR spectrum of the Ga(III)-photoxenobactin E tautomers (**2A/B**) (DMSO-d_6, 500 MHz, 298 K). S10

Figure S8. ^{1}H,^{1}H-NOESY NMR spectrum of the Ga(III)-photoxenobactin E tautomers (**2A/B**) (DMSO-d_6, 500 MHz, 298 K). S10

Figure S9. ^{1}H NMR spectrum of the Ga(III)-photoxenobactin E tautomer (**2T**) (DMSO-d_6, 400 MHz, 348.15 K). S11

Figure S10. (A) Dynamic ^{1}H NMR of the region associated with the methylene resonances of H-16 of Ga(III)-photoxenobactin E tautomers (2A/B) (DMSO-d_6, 400 MHz, 298.15, 313.15 and 348.15 K) and their structures at 298.15 K (2A/B) and at 348.15 K (2T). (B) Selective TOCSY irradiation experiment at δ_H 3.23 of the Ga(III)-photoxenobactin E (2T) (DMSO-d_6, 400 MHz, 348.15 K); (C) ^{1}H,^{1}H-COSY NMR of Ga(III)-photoxenobactin E (**2**) (DMSO-d_6, 400 MHz, 348.15 K); (D) ^{1}H,^{13}C-HSQC NMR spectrum of Ga(III)-photoxenobactin E (**2**) (DMSO-d_6, 500 MHz, 298.15 K); (E) ^{1}H,^{13}C-HSQC NMR spectrum of Ga(III)-photoxenobactin E (2) (DMSO-d_6, 400 MHz, 348.15 K). S12

B – NMR experiments obtained in DMSO-d_6:CD_3OD 9:1 mixture. *S13*

Figure S11. Variable temperature ^{1}H NMR spectra of the tautomeric mixture of the Ga(III)-photoxenobactin E complex (**2A/B**) measured in DMSO-d_6:CD_3OD (9:1) from 293.15 to 323.15 K (400 MHz). S13

C – NMR experiments obtained in $CDCl_3$. *S14*

Figure S12. ^{1}H NMR spectrum of the Ga(III)-photoxenobactin E tautomers (**2A/B**) ($CDCl_3$, 500 MHz, 298 K). S14

Figure S13. ^{1}H,^{1}H-COSY NMR spectrum of the Ga(III)-photoxenobactin E tautomers (**2A/B**) ($CDCl_3$, 500 MHz, 298 K). S14

Figure S14. ^{1}H,^{13}C-HSQC NMR spectrum of the Ga(III)-photoxenobactin E tautomers (**2A/B**) ($CDCl_3$, 500 MHz, 298 K). S15

Figure S15. ^{1}H,^{13}C-HMBC NMR spectrum of the Ga(III)-photoxenobactin E tautomers (**2A/B**) ($CDCl_3$, 500 MHz, 298 K). S15

D – NMR experiments obtained in THF-d_8. .... *S16*

Figure S16. ^{1}H NMR spectrum of Ga(III)-photoxenobactin E tautomers (**2A/B**) THF-d_8, 500 MHz, 298 K). S16

Figure S17. ^{1}H,^{1}H-COSY NMR spectrum of Ga(III)-photoxenobactin E tautomers (**2A/B**) (THF-d_8, 500 MHz, 298 K). S16

Figure S18. $^{1}H,^{13}C$-HSQC NMR spectrum of Ga(III)-photoxenobactin E tautomers (**2A/B**) (THF-d_8, 500 MHz, 298 K). S17

Figure S19. $^{1}H,^{13}C$-HMBC NMR spectrum of Ga(III)-photoxenobactin E tautomers (**2A/B**) (THF-d_8, 500 MHz, 298 K). S17

E – DFT Calculations. *S18*

Ga(III)-photoxenobactin E, thiol-form: S18

Figure S20. Ball and sticks model of Ga(III)-photoxenobactin E thiol tautomer. S18

Table S1. DFT data of the WB97XD/DEF2TVPP model Ga(III)-photoxenobactin E thiol tautomer. S18

Ga(III)-Photoxenobactin E, thione-form: S21

Figure S21. Ball and sticks model of Ga(III)-photoxenobactin E thione tautomer S21

Table S2. DFT data of the WB97XD/DEF2TVPP model Ga(III)-photoxenobactin E thione tautomer. S21

References *S24*

General experimental procedures. ^{1}H, ^{13}C, and 2D NMR spectra were recorded on a Bruker Avance 500 (500 MHz for ^{1}H and 125 MHz for ^{13}C) with a dual cryoprobe or a Bruker Avance 400 (400 MHz for ^{1}H and 100 MHz for ^{13}C) with a BBI probe. DMSO-d_6, CD_3OD, $CDCl_3$ and THF-d_8 were used as deuterated solvents. Chemical shifts are reported in δ scale relative to DMSO-d_6 (δ 2.50 ppm for ^{1}H NMR, δ 39.5 ppm for ^{13}C NMR), CD_3OD (δ 3.31 ppm for ^{1}H NMR, δ 49.0 ppm for ^{13}C NMR), $CDCl_3$ (δ 7.26 ppm for ^{1}H NMR, δ 77.5 ppm for ^{13}C NMR) and THF-d_8 (δ 3.58 ppm for ^{1}H NMR, δ 25.4 or δ 67.6 ppm for ^{13}C NMR). HPLC separation was performed on an Agilent 1100 or 1200 using reversed-phase chromatographic columns. HRMS data were acquired on a LTQ-Orbitrap Discovery mass spectrometer coupled to a Accela HPLC (Thermo Fischer Scientific). Infrared spectra were recorded on a Nicolet iS10 FTIR Spectrometer (Thermo Fisher Scientific).

Bacterial cultivation and siderophore isolation. *Vibrio anguillarum* RV22 grown in CM9 supplemented with 2,2′-dipyridyl (25 μM) was used to obtain cell-free supernatants by centrifugation (Beckman J-21 High Speed Centrifuge)[1]. The bacterial culture was incubated at 15 °C with shaking at 150 rpm to achieve a $OD_{600} \approx 0.8$. A 1:50 dilution of a *V. anguillarum* overnight culture grown in Tryptic Soy Broth (Condalab, Madrid, Spain) to an $OD_{600} = 0.5$ was used as inoculum. Two liters of cell-free supernatants were extracted following the protocol described by Souto et al,[2] where the supernatant was concentrated to 300 mL under reduced pressure and subsequently chelated with 0.1 mM gallium(III)-acetylacetonate ($(C_5H_8O_2)_3Ga$). The supernatant was fractionated in three rounds by loading 100 mL onto a preconditioned OASIS® HLB cartridge (35cc, 6 g) and eluted with 60 mL of the following mixtures of H_2O:CH_3CN: 1:0 (H1), 7:3 (H2), 1:1 (H3), 3:7 (H4), 0:1 (H5). The fractions were profiled via HPLC-DAD-MS using an Atlantis dC18 (100 x 4.6 mm, 5 μm, Waters) column with a mobile phase consisting of 20% to 90% CH_3CN in H_2O (v/v) over 35 min, then 90% CH_3CN isocratic over 5 min, at a flow rate of 0.5 mL/min. Mass spectra were acquired in positive mode, with resolution set to 30,000 in the range of *m/z* 150–1500. MS parameters were set as follows: spray voltage of 3.90 kV, capillary temperature of 350°C, sheath gas rate of 50 units N_2 (ca. 500 mL/min), and auxiliary gas rate of 10 units N_2 (ca. 100 mL/min). The maximum ion injection time was set to 100 ms. In fraction H3 we detected the known Ga(III)-piscibactin complex[2] and an ion with a mass corresponding to the Ga(III)-photoxenobactin E complex (HRESIMS *m/z* 535.9720 $[M + H]^+$). Fraction H3 was subjected to RP-HPLC (Agilent 1100 or 1200) on a semi-preparative

Atlantis dC18 column (100 x 10 mm, 5 µm, Waters) with a gradient of CH_3CN in H_2O from 20:80 to 45:55 over 30 min at a flow rate of 1.5 mL/min to yield 2.4 mg of Ga(III)-photoxenobactin (**2**) (t_R 23.8 min).

NMR analysis. ^1H, ^{13}C, and 2D NMR spectra were recorded on a Bruker Avance 500 with a dual cryoprobe or a Bruker Avance 400 with a BBI probe. A Bruker BSVT smart multichannel variable temperature control unit was used in both, the isothermal and variable temperature NMR (VT NMR) experiments, to control the temperature of the cooling dry air stream. For variable-temperature experiments, the NMR probe temperature was calibrated using the proton-shift of methanol. When not stated explicitly, the probe temperature was set to 298.15 K. Dynamic ^1H NMR line shape simulations were performed using WinDNMR free-ware, Version 7.1.13, Hans J. Reich, Wisconsin, WI, USA.[3] Simulations were performed for two CH_3-18 singlets observed for the different tautomers (temperature range 293-323 K). The relative molar fractions were determined at each temperature by integrating the proton resonances of CH_3-18. The rate constants were estimated at each temperature by superimposing the experimental spectra and those simulated in WinDNMR. The simulation provided the value of $k_{2B \rightarrow 2A} + k_{2A \rightarrow 2B}$ which was multiplied by the molar fraction of **2A** to obtain the rate of exchange. The corresponding Eyring plot ($\ln(k_{ex}/T)$ vs $(1/T)$) was used to estimate the activation enthalpy ($\Delta H^{\ddagger}$) and activation entropy ($\Delta S^{\ddagger}$).

DFT calculations. The structure of Ga(III)-photoxenobactin E was minimized firstly by a conformational search performed by employing MAESTRO software, using an energy window of 5 kcal/mol. Minimized energy was done at the WB97XD/DEF2TVPP level, solvent model = IEFPCM (DMSO), the vibrational frequency calculations confirming the nature of the optimized geometries as local energy minima, using the Gaussian16 program. Chemical shielding tensors (CST) were computed at the GIAO/MPW1PW91/6-311++G(2d,p) (solvent model = IEFPCM (DMSO)). Tantillo's slope and intercept of -1.0490 and 186.6525 respectively were used to correct calculated carbon chemical shifts.[4]

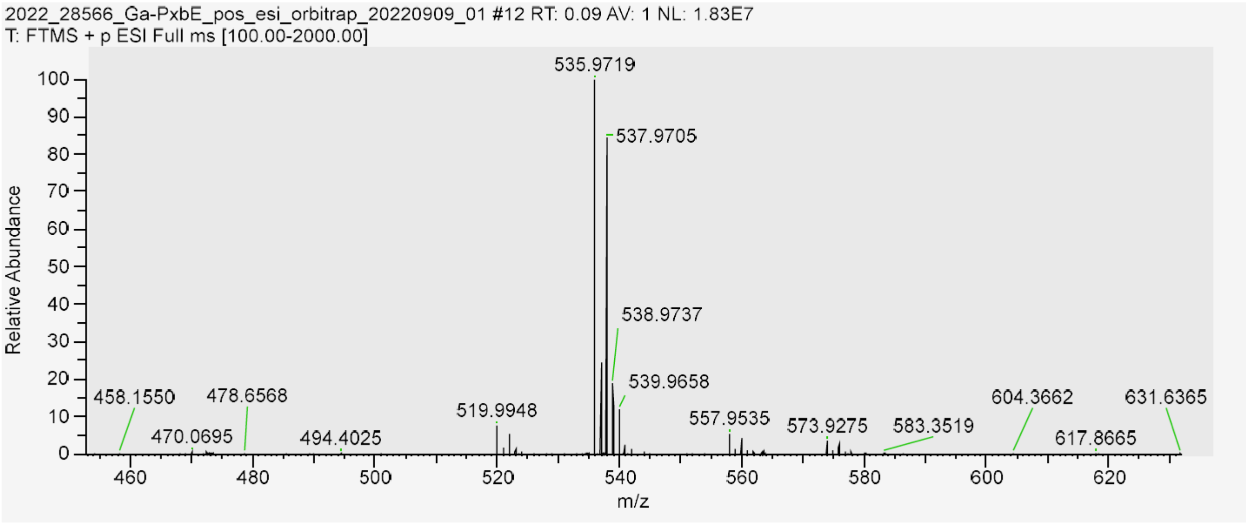

Figure S1. HRESIMS spectrum of Ga(III)-photoxenobactin E (**2**) isolated from *V. anguillarum* RV22.

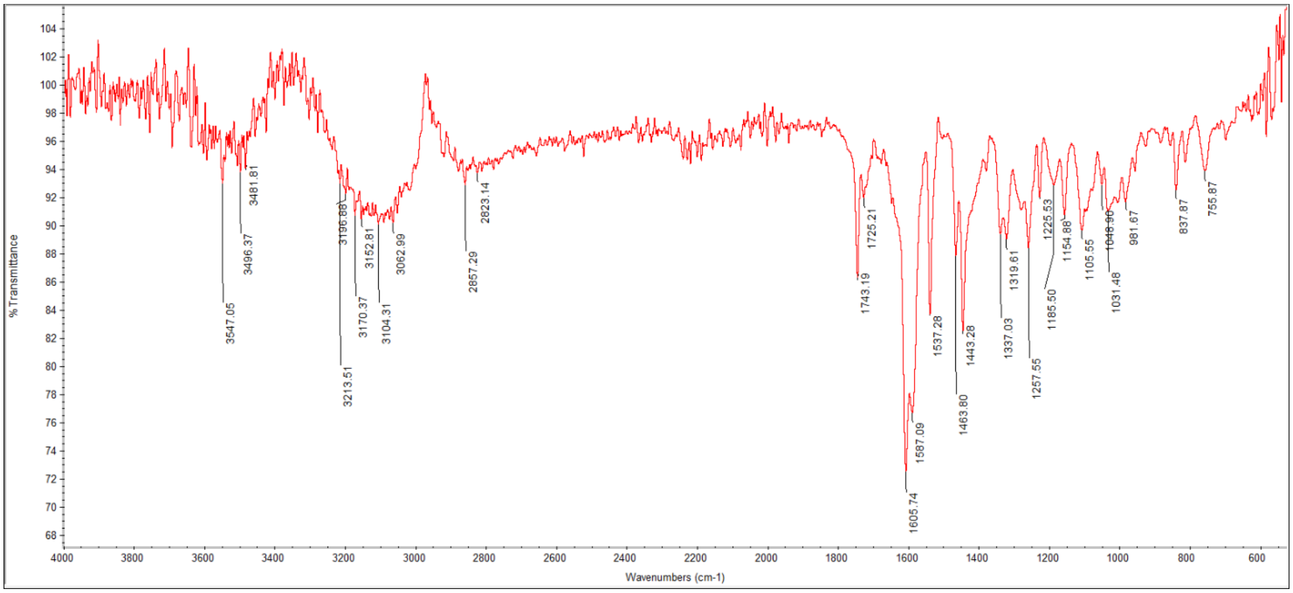

Figure S2. IR spectrum of Ga(III)-photoxenobactin E tautomers (**2A/B**) in EtOAc (%Transmittance)

.

A – NMR experiments obtained in DMSO-d_6.

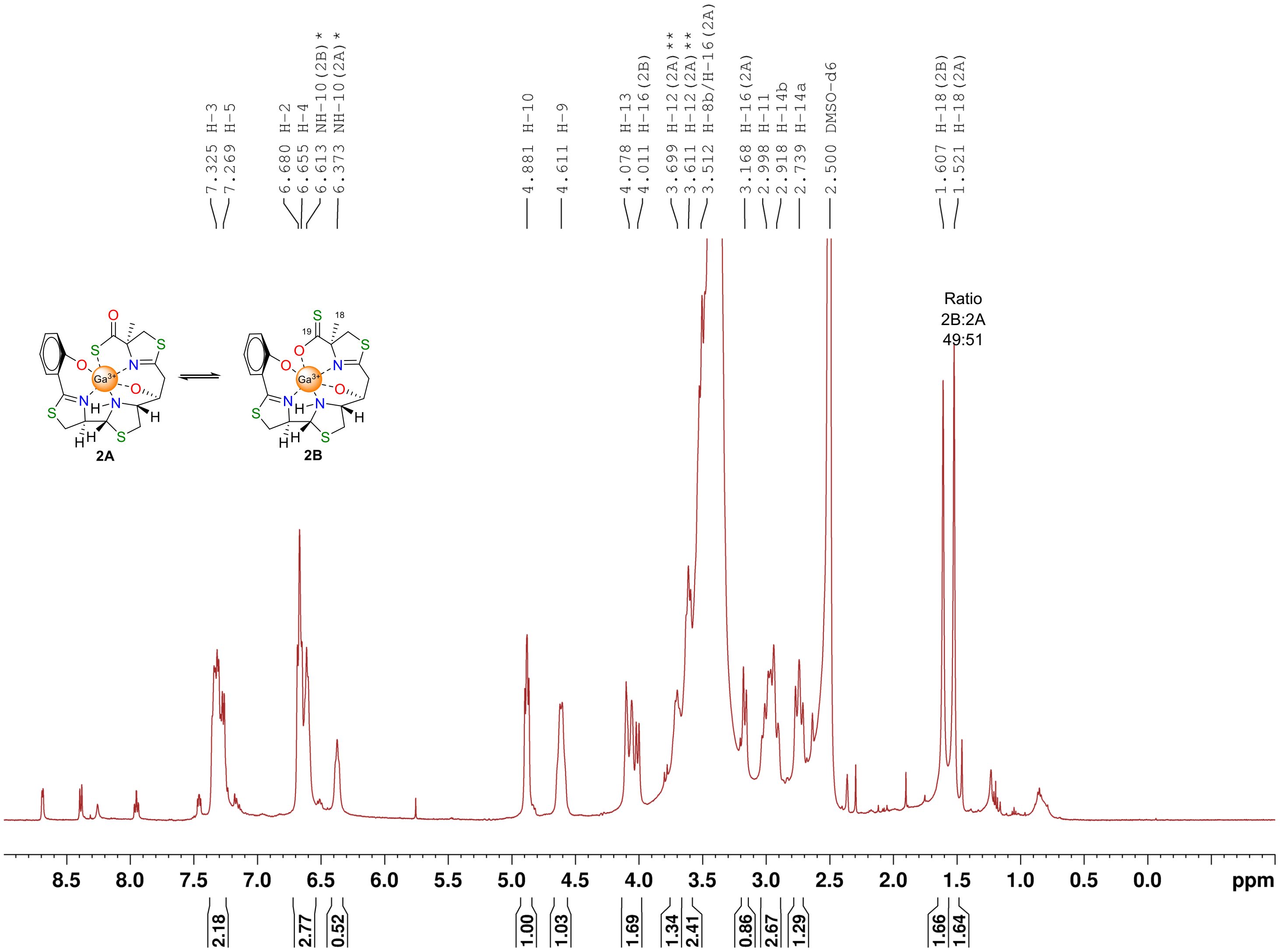

Figure S3. ^{1}H NMR spectrum of the Ga(III)-photoxenobactin E tautomers (**2A/B**) (DMSO-d_6, 500 MHz, 298 K). *, ** = interchangeable signals

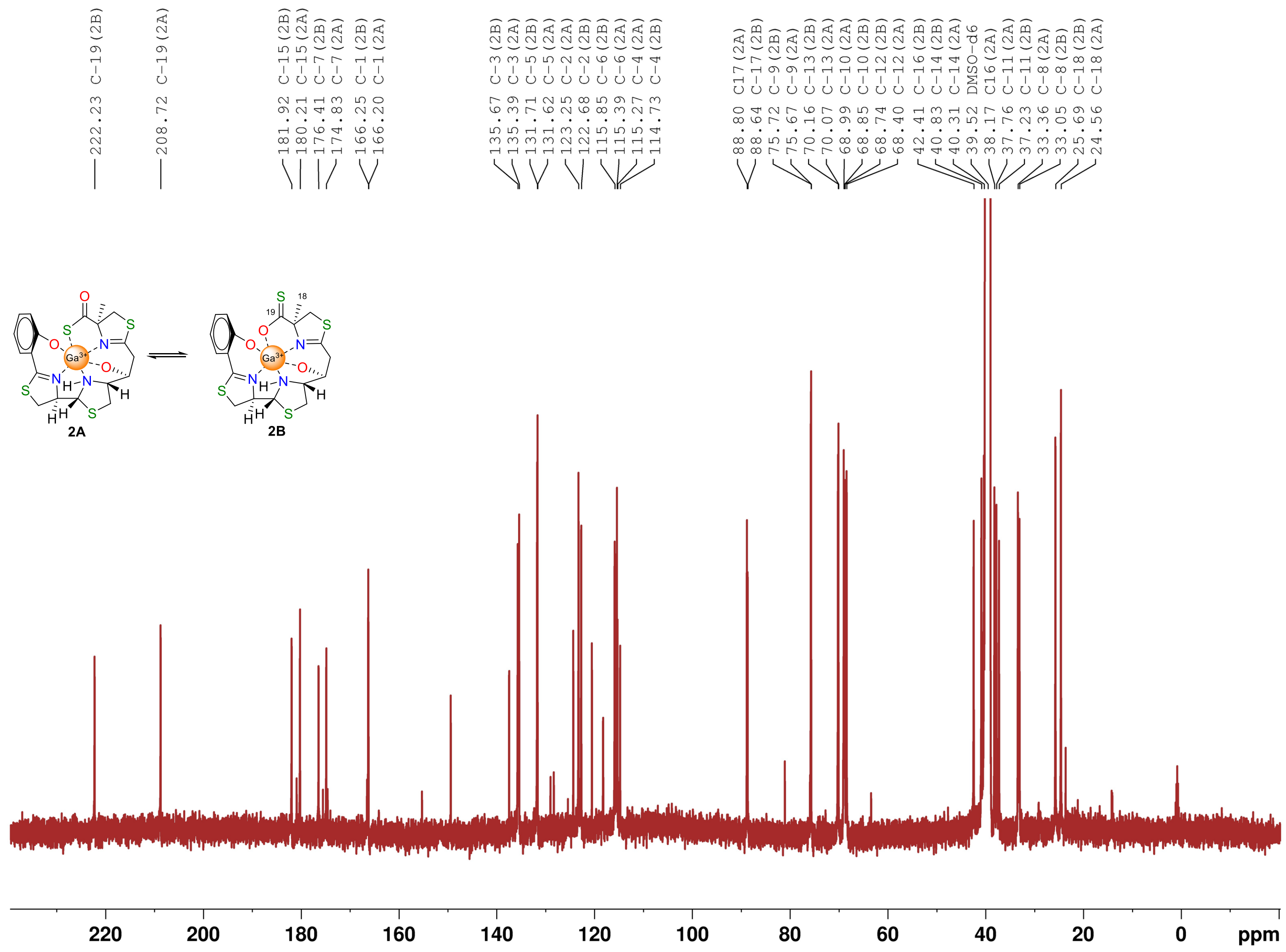

Figure S4. ^{13}C NMR spectrum of the Ga(III)-photoxenobactin E tautomers (**2A/B**) (DMSO-d_6, 125 MHz, 298 K).

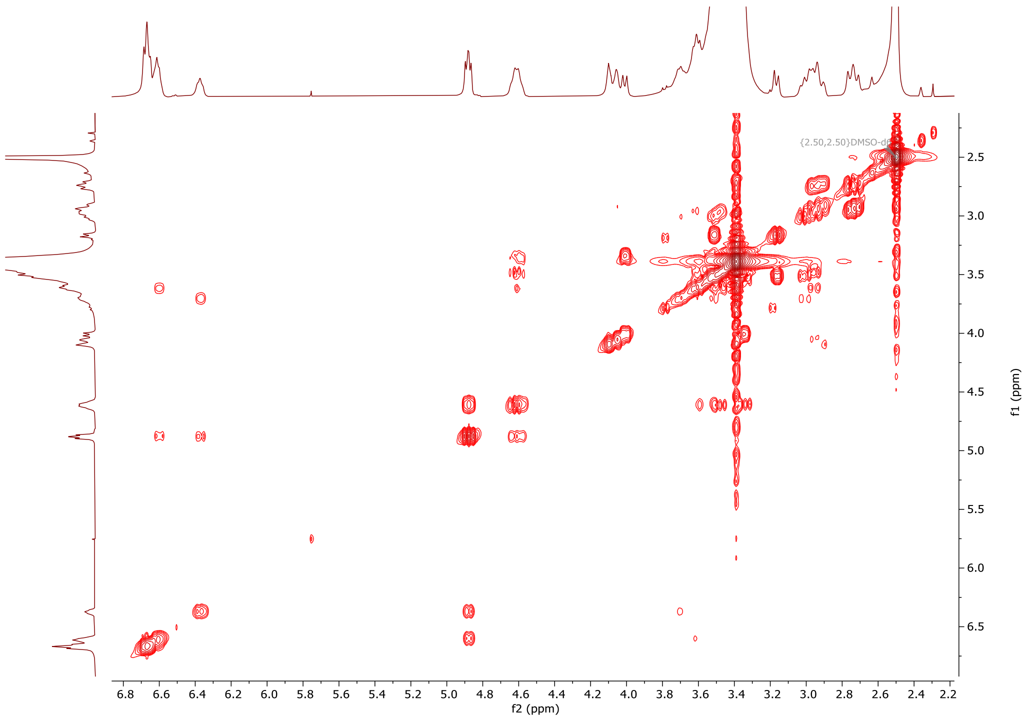

Figure S5. $^{1}H,^{1}H$-COSY NMR spectrum of the Ga(III)-photoxenobactin E tautomers (**2A/B**) (DMSO-d_6, 500 MHz, 298 K).

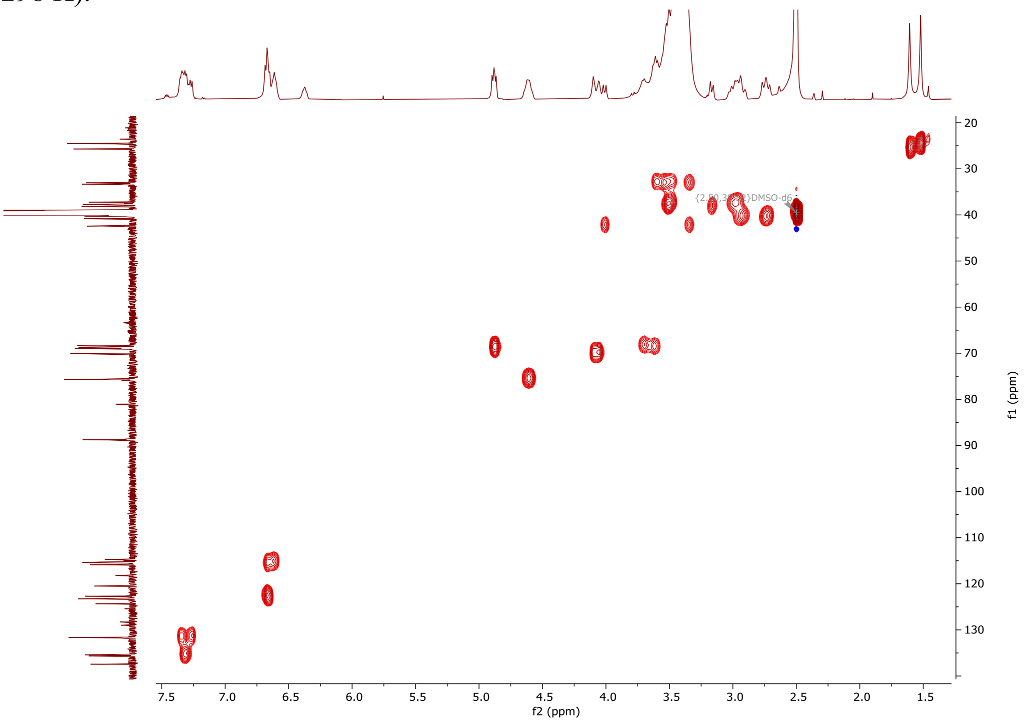

Figure S6. $^{1}H,^{13}C$-HSQC NMR spectrum of the Ga(III)-photoxenobactin E tautomers (**2A/B**) (DMSO-d_6, 500 MHz, 298 K)

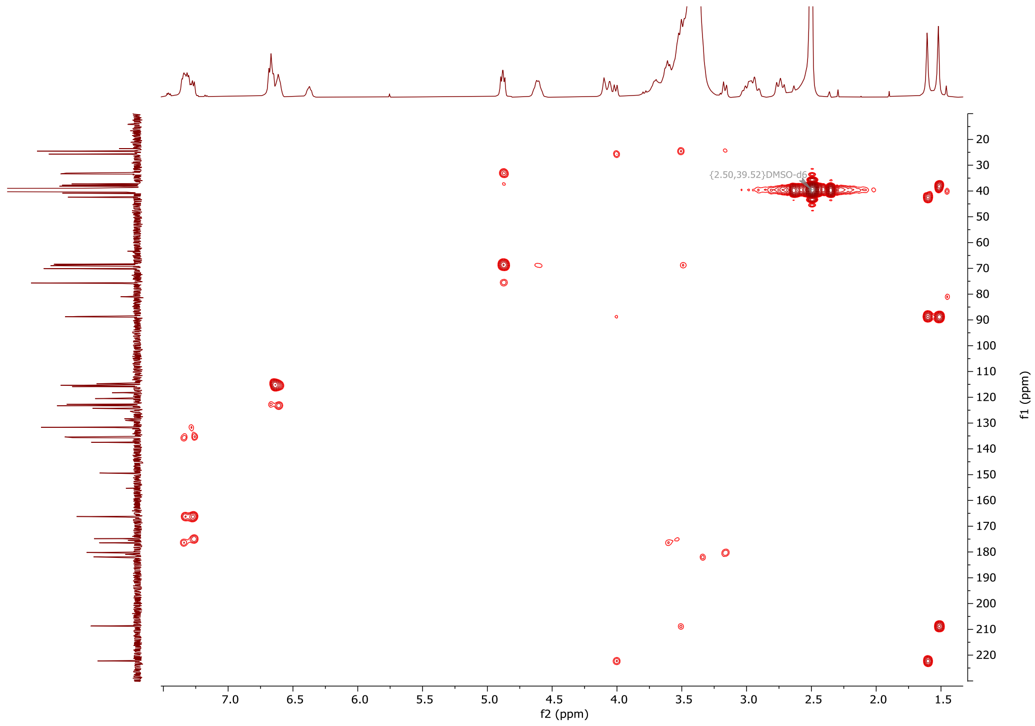

Figure S7. ^{1}H,^{13}C-HMBC NMR spectrum of the Ga(III)-photoxenobactin E tautomers (**2A/B**) (DMSO-d_6, 500 MHz, 298 K).

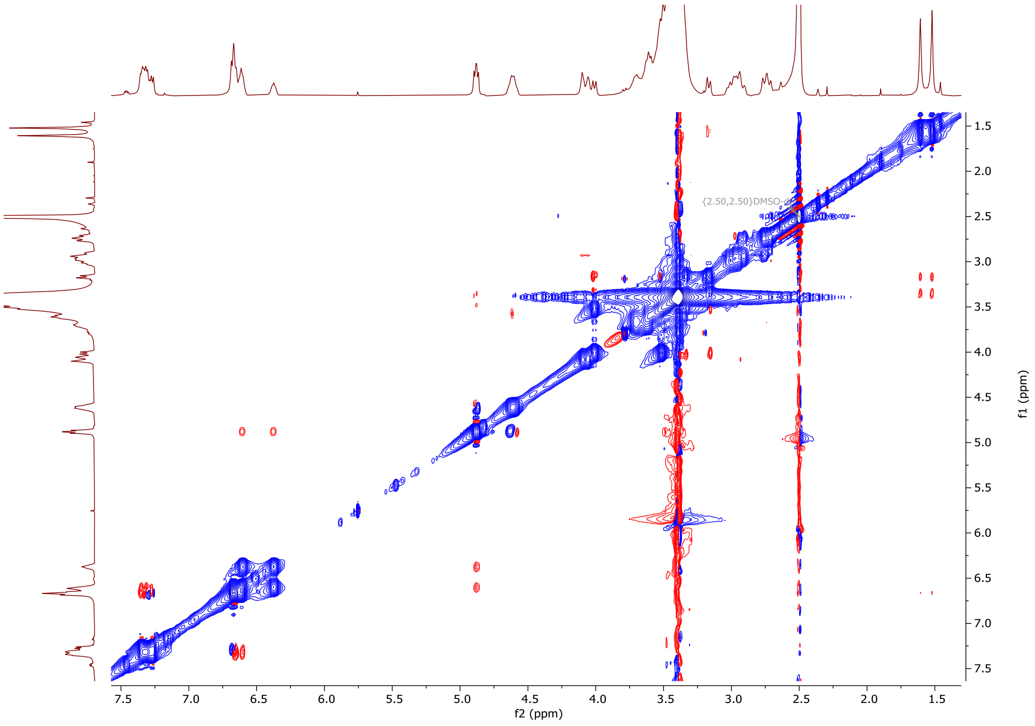

Figure S8. ^{1}H,^{1}H-NOESY NMR spectrum of the Ga(III)-photoxenobactin E tautomers (**2A/B**) (DMSO-d_6, 500 MHz, 298 K).

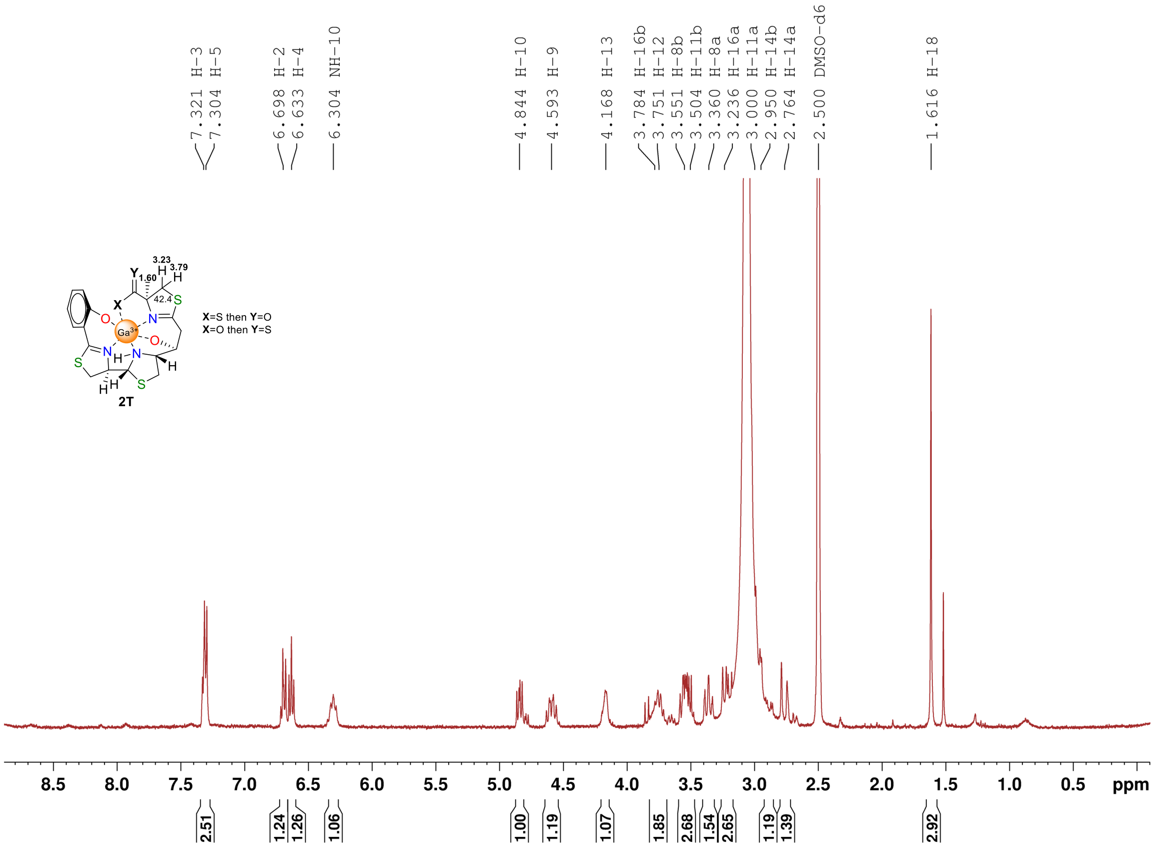

Figure S9. ^{1}H NMR spectrum of the Ga(III)-photoxenobactin E tautomer (**2T**) (DMSO-d_6, 400 MHz, 348.15 K).

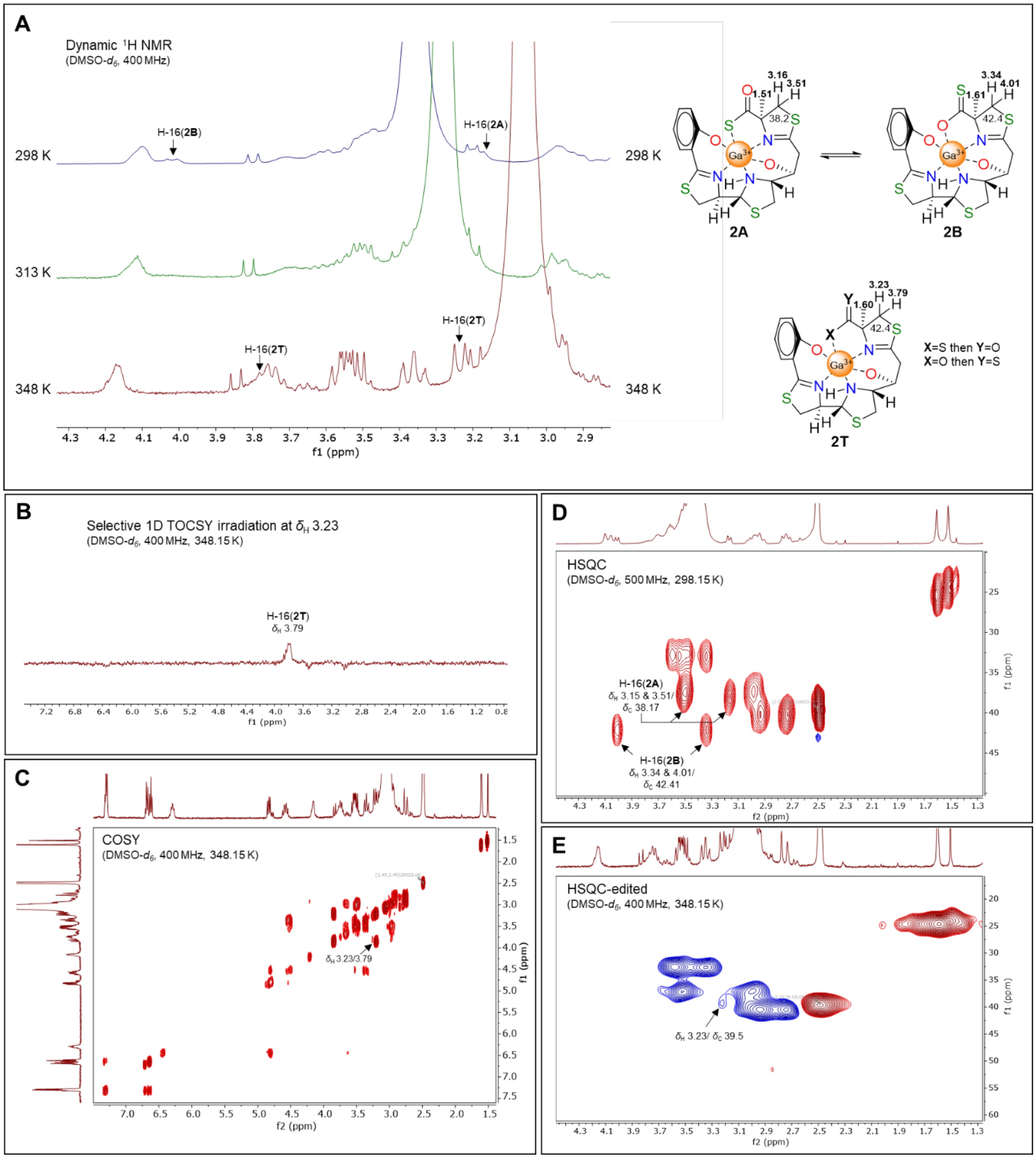

Figure S10. (A) Dynamic ^{1}H NMR of the region associated with the methylene resonances of H-16 of Ga(III)-photoxenobactin E tautomers (**2A/B**) (DMSO-d_6, 400 MHz, 298.15, 313.15 and 348.15 K) and their structures at 298.15 K (**2A/B**) and at 348.15 K (**2T**). (B) Selective TOCSY irradiation experiment at δ_H 3.23 of the Ga(III)-photoxenobactin E (**2T**) (DMSO-d_6, 400 MHz, 348.15 K); (C) ^{1}H,^{1}H-COSY NMR of Ga(III)-photoxenobactin E (**2**) (DMSO-d_6, 400 MHz, 348.15 K); (D) ^{1}H,^{13}C-HSQC NMR spectrum of Ga(III)-photoxenobactin E (**2**) (DMSO-d_6, 500 MHz, 298.15 K); (E) ^{1}H,^{13}C-HSQC NMR spectrum of Ga(III)-photoxenobactin E (**2**) (DMSO-d_6, 400 MHz, 348.15 K).

B – NMR experiments obtained in DMSO-d_6:CD_3OD 9:1 mixture.

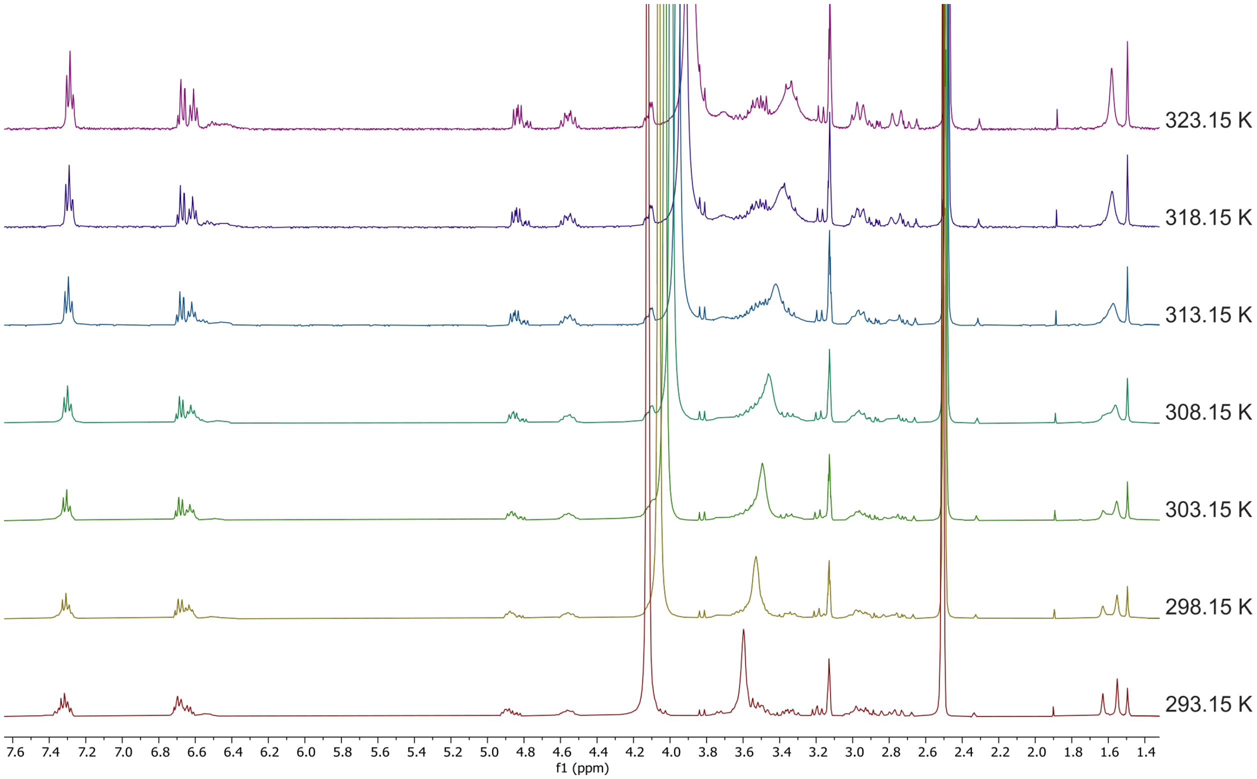

Figure S11. Variable temperature ^{1}H NMR spectra of the tautomeric mixture of the Ga(III)-photoxenobactin E complex (**2A/B**) measured in DMSO-d_6:CD_3OD (9:1) from 293.15 to 323.15 K (400 MHz).

C – NMR experiments obtained in CDCl₃.

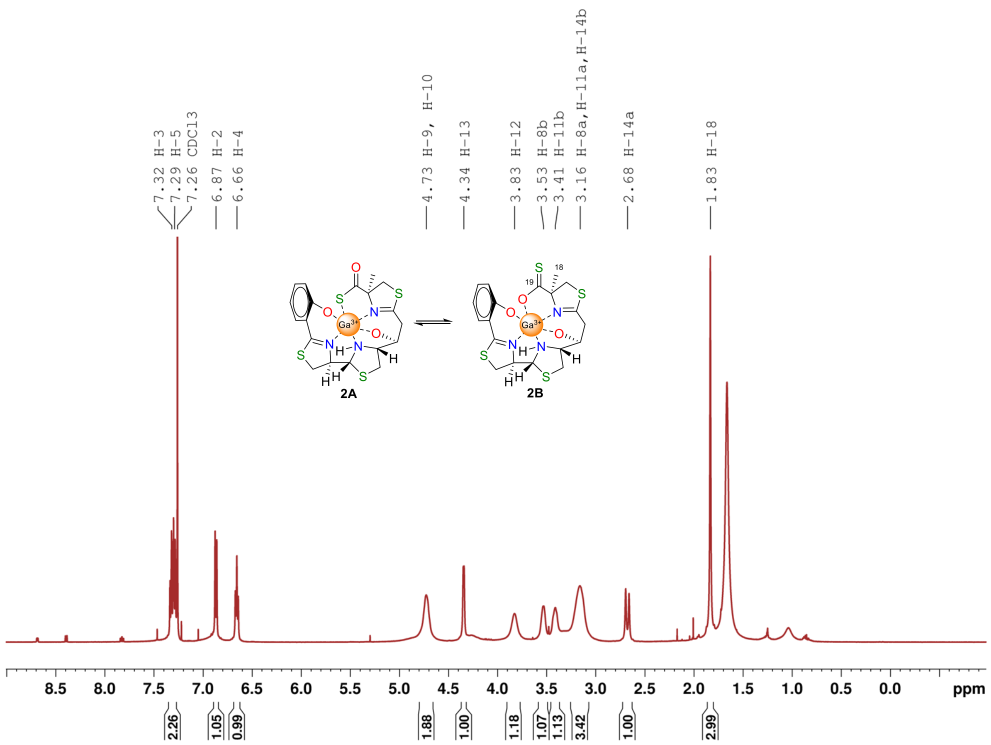

Figure S12. ^{1}H NMR spectrum of the Ga(III)-photoxenobactin E tautomers (**2A/B**) ($CDCl_3$, 500 MHz, 298 K).

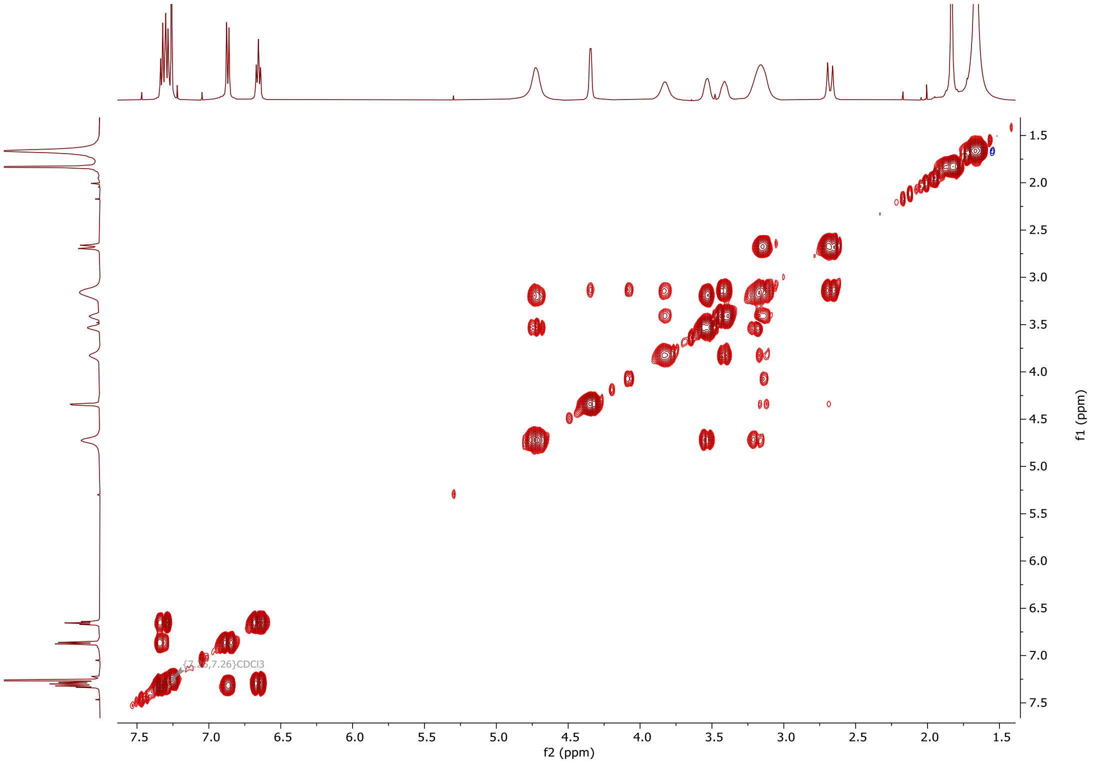

Figure S13. $^{1}H,^{1}H$-COSY NMR spectrum of the Ga(III)-photoxenobactin E tautomers (**2A/B**) ($CDCl_3$, 500 MHz, 298 K).

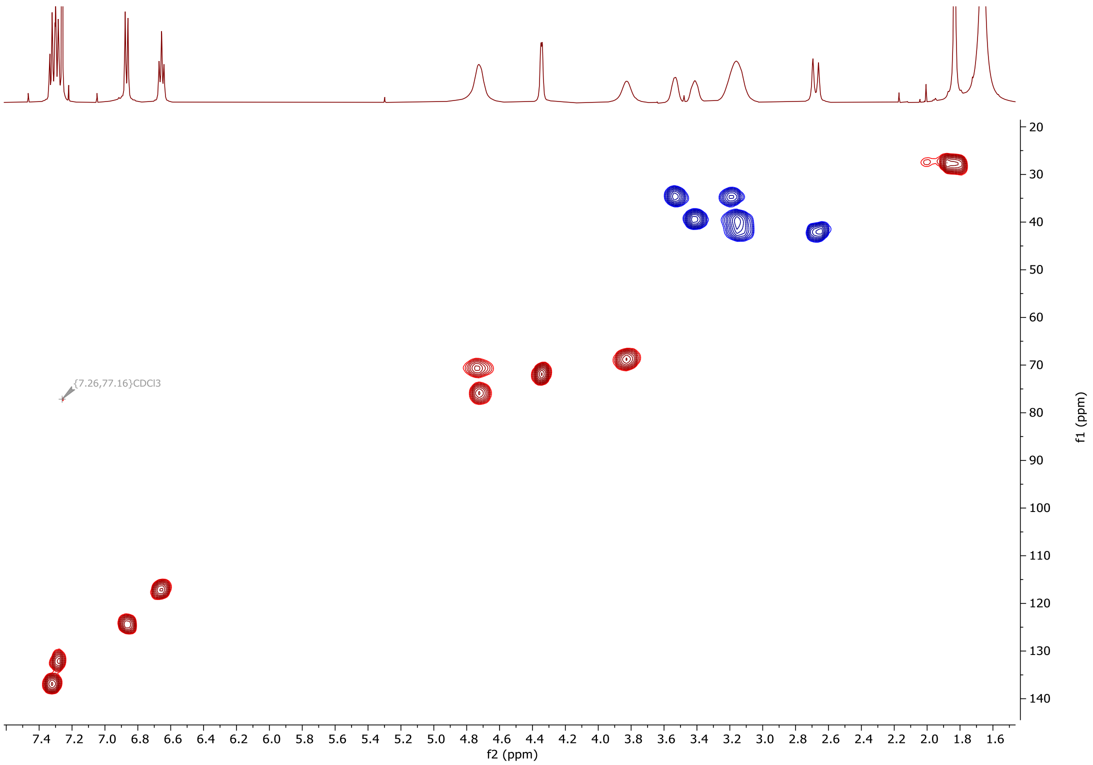

Figure S14. ^{1}H,^{13}C-HSQC NMR spectrum of the Ga(III)-photoxenobactin E tautomers (**2A/B**) ($CDCl_3$, 500 MHz, 298 K).

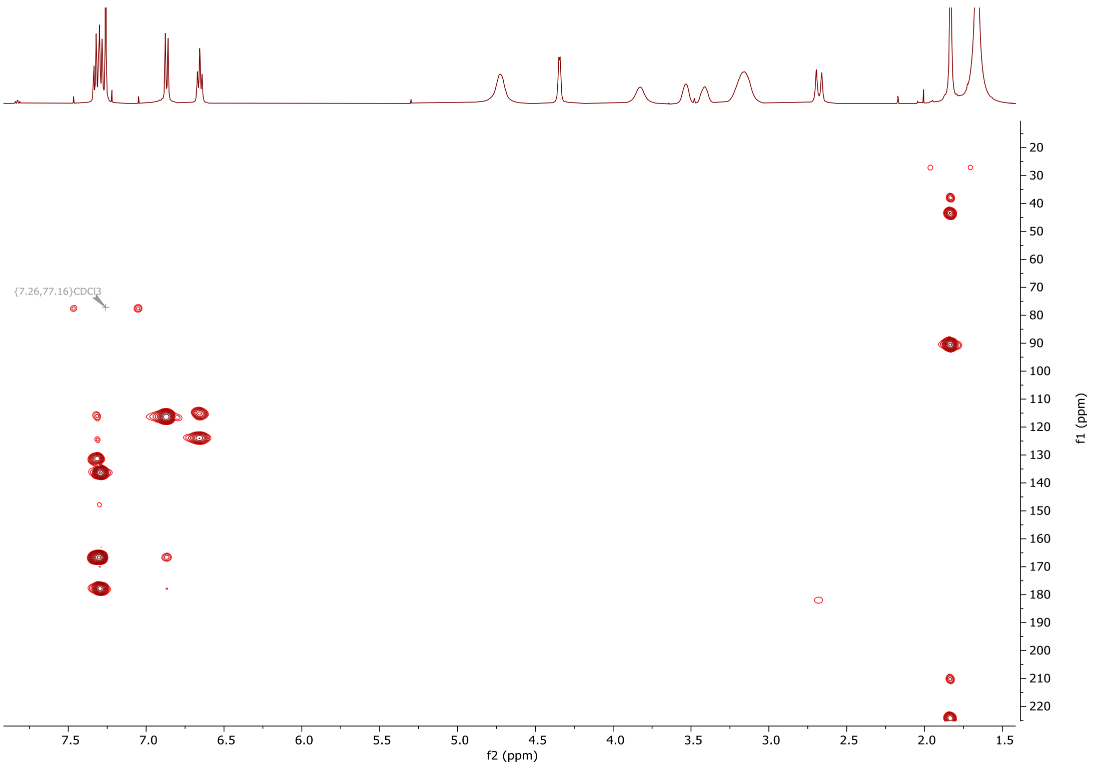

Figure S15. ^{1}H,^{13}C-HMBC NMR spectrum of the Ga(III)-photoxenobactin E tautomers (**2A/B**) ($CDCl_3$, 500 MHz, 298 K).

D – NMR experiments obtained in THF-d_8.

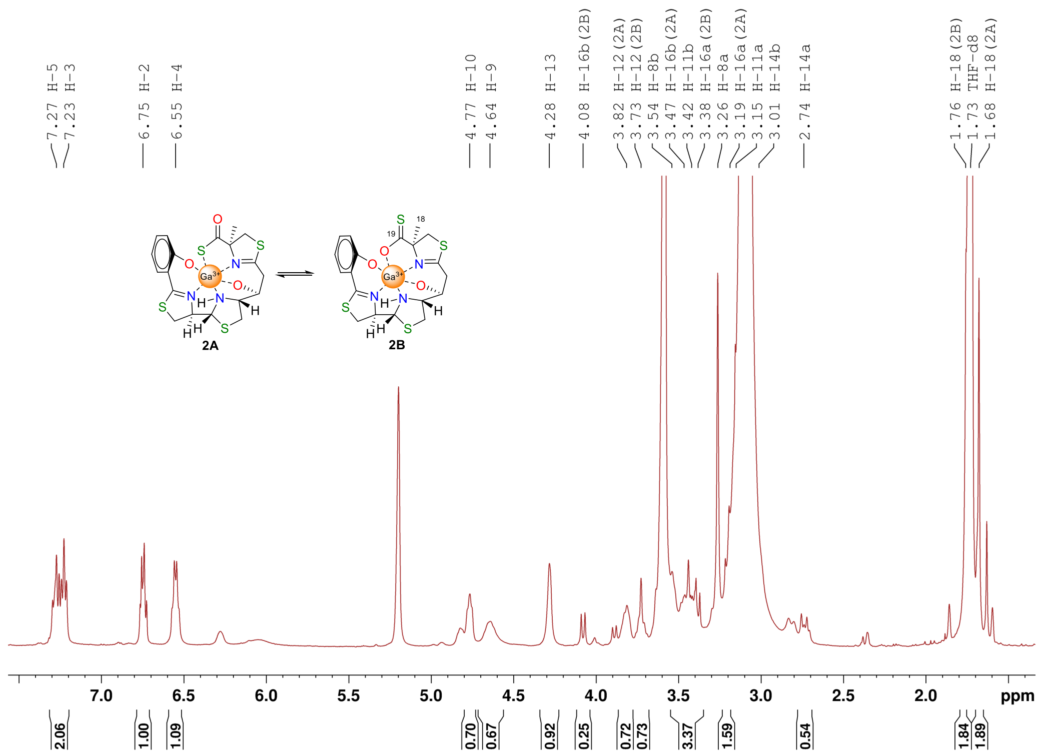

Figure S16. ^{1}H NMR spectrum of Ga(III)-photoxenobactin E tautomers (**2A/B**) THF-d_8, 500 MHz, 298 K).

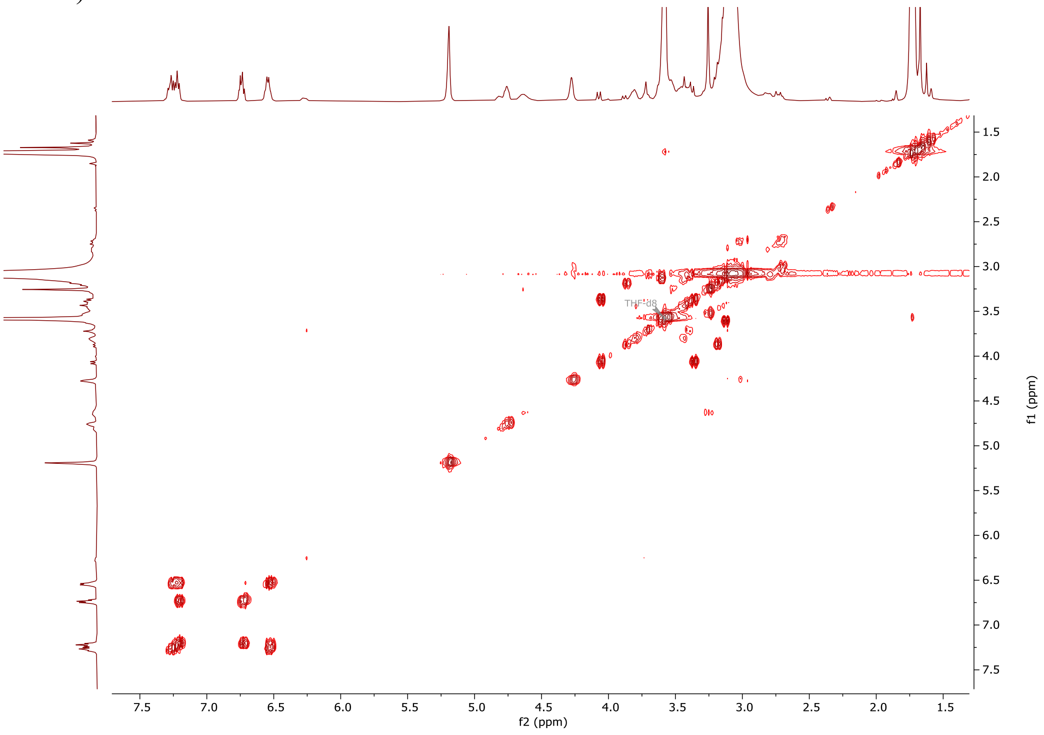

Figure S17. ^{1}H,^{1}H-COSY NMR spectrum of Ga(III)-photoxenobactin E tautomers (**2A/B**) (THF-d_8, 500 MHz, 298 K).

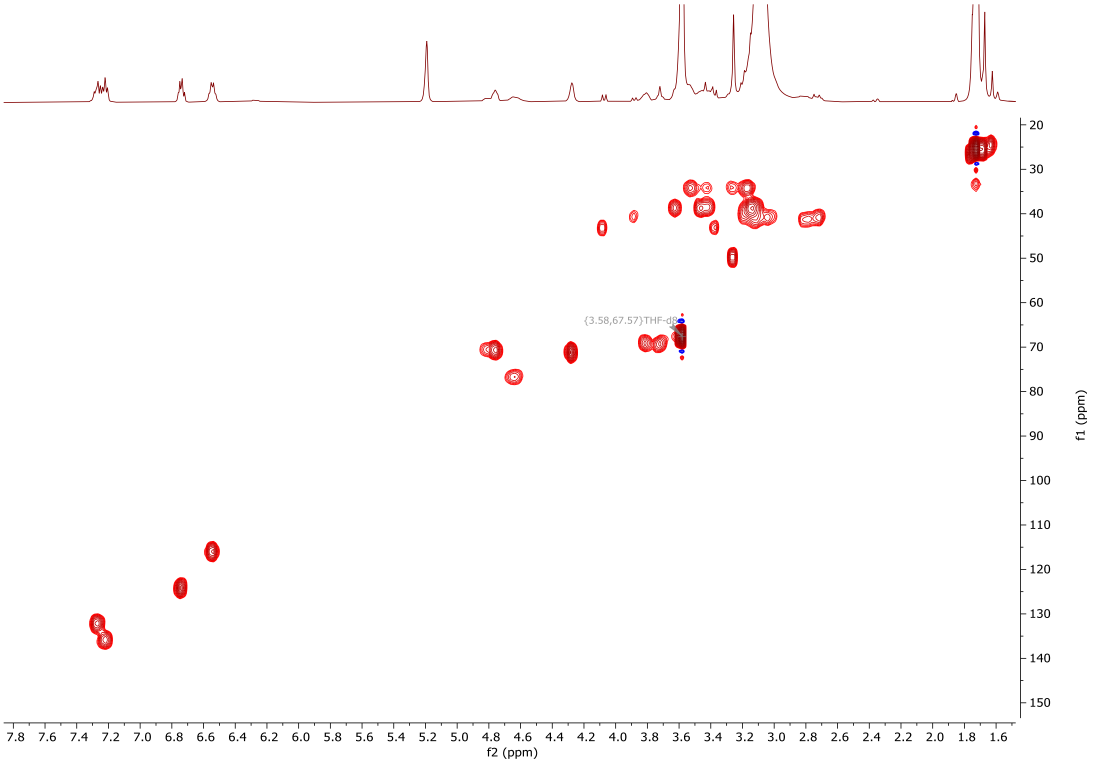

Figure S18. $^{1}H,^{13}C$-HSQC NMR spectrum of Ga(III)-photoxenobactin E tautomers (**2A/B**) (THF-d_8, 500 MHz, 298 K).

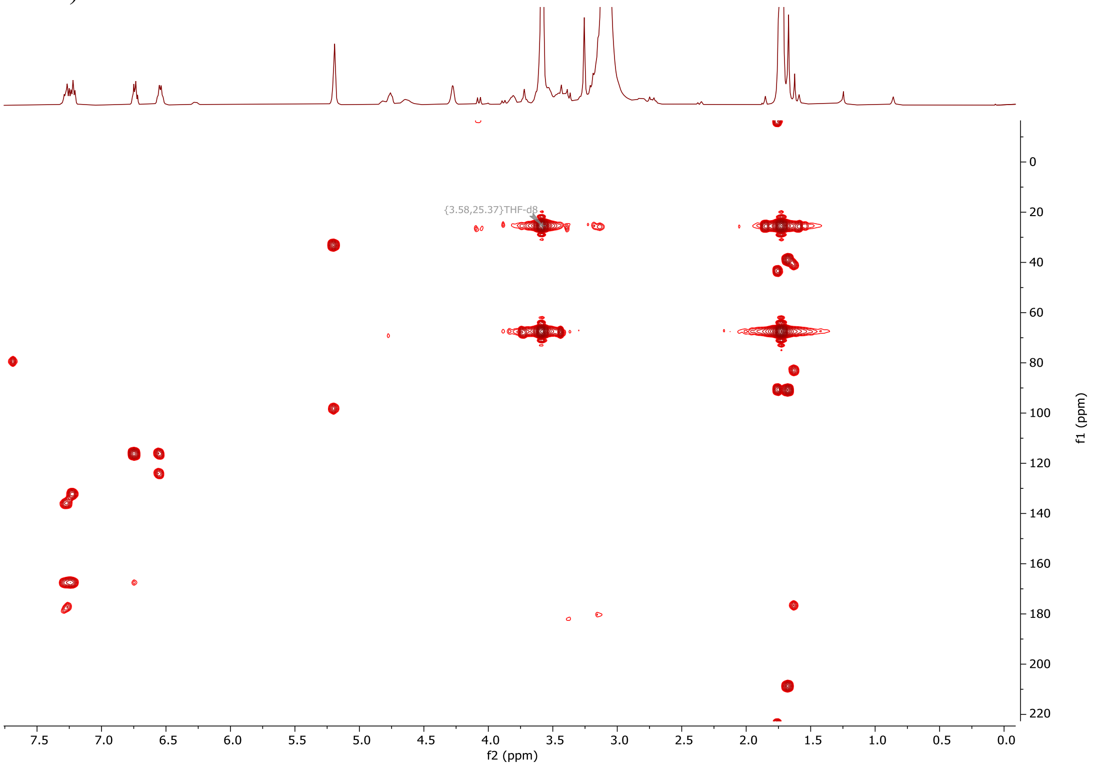

Figure S19. $^{1}H,^{13}C$-HMBC NMR spectrum of Ga(III)-photoxenobactin E tautomers (**2A/B**) (THF-d_8, 500 MHz, 298 K).

E – DFT Calculations.

Tautomers of Ga(III)-Photoxenobactin E were minimized by a conformational search performed by employing MAESTRO software, using an energy window of 5 kcal/mol. Minimized energy/frequency was done at WB97XD/DEF2TVPP level, solvent model = IEFPCM (DMSO) Chemical shielding tensors (CST) were computed at the GIAO/MPW1PW91/6-311++G(2d,p) (solvent model = IEFPCM (DMSO)). Tantillo's slope and intercept of -1.0490 and 186.6525 respectively were used to correct calculated carbon chemical shifts.

Ga(III)-photoxenobactin E, thiol-form:

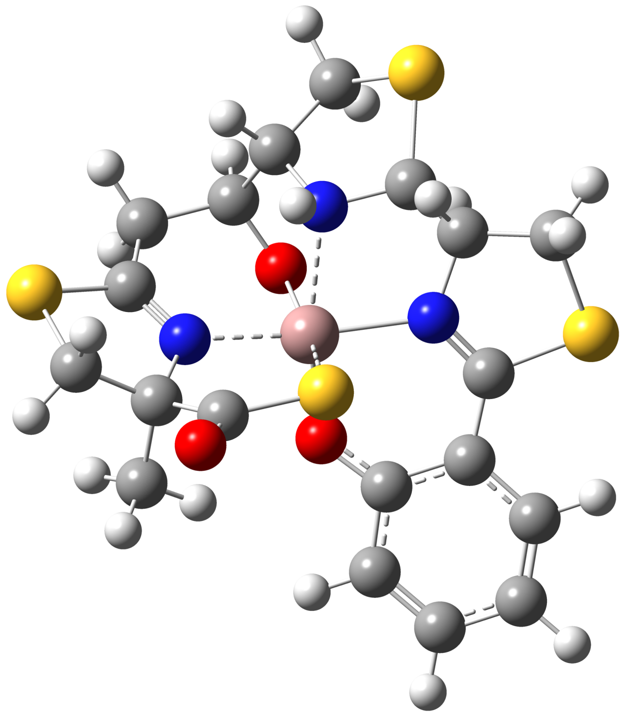

Figure S20. Ball and sticks model of Ga(III)-photoxenobactin E thiol tautomer.

Table S1. DFT data of the WB97XD/DEF2TVPP model Ga(III)-photoxenobactin E thiol tautomer.

Zero-point correction= 0.380967 (Hartree/Particle)
Thermal correction to Energy= 0.407157
Thermal correction to Enthalpy= 0.408101
Thermal correction to Gibbs Free Energy= 0.325584
Sum of electronic and zero-point Energies= -4643.592395
Sum of electronic and thermal Energies= -4643.566206
Sum of electronic and thermal Enthalpies= -4643.565261
Sum of electronic and thermal Free Energies= -4643.647779

Number of Imaginary Frequencies = 0

Coordinates:

Symbol	X	Y	Z
C	2.9641640	-1.2856430	-0.3148840
C	1.9011190	-2.0368440	-0.8998440
C	2.1730700	-3.3818240	-1.2305540
C	3.3964280	-3.9564910	-0.9868270
C	4.4306840	-3.2201670	-0.4013420
C	4.2033150	-1.9053090	-0.0803260
C	2.8064850	0.1192350	0.0014730
S	4.1766550	1.1676620	0.3455750
C	3.0502850	2.4912500	0.8767120
C	1.7220510	2.1591750	0.1992200
N	1.6740320	0.7228250	0.0191820
O	0.7144710	-1.5703370	-1.1737770
H	1.3711920	-3.9519110	-1.6806740
H	3.5568470	-4.9947840	-1.2484180
H	5.3902940	-3.6757160	-0.2038060
H	4.9961740	-1.3281380	0.3788100
H	3.4477750	3.4513460	0.5641880
H	2.9712930	2.4668550	1.9626270
H	1.6898860	2.6067960	-0.7978780
Ga	-0.1400460	-0.0781830	-0.3190970
C	0.4853830	2.6108190	0.9945800
S	0.1410410	4.3758630	0.7489220
C	-0.7359890	3.9410210	-0.7710870
C	-1.4774640	2.6312630	-0.4825830
N	-0.7205040	1.8922740	0.5572970
H	0.6454230	2.4141050	2.0529350
H	-0.0282290	3.8150980	-1.5898010
H	-1.4251760	4.7399070	-1.0302790
H	-2.4629230	2.8645530	-0.0821230
H	-1.3080820	1.7395160	1.3645660

C	-1.5990610	1.7532590	-1.7648300
C	-2.9292740	0.9786680	-1.8330480
C	-3.0899500	-0.1201540	-0.8346420
S	-4.6857410	-0.6788630	-0.4280400
C	-4.0110040	-1.7290730	0.8846510
C	-2.5044960	-1.8571150	0.5793240
N	-2.1197680	-0.7241620	-0.2613520
C	-2.1905250	-3.1490400	-0.1840560
H	-2.7932850	-3.1923260	-1.0918570
H	-2.4256620	-4.0113720	0.4376560
H	-1.1377590	-3.1758580	-0.4614590
C	-1.7020360	-1.8785910	1.8962720
O	-2.1549510	-2.5044260	2.8325630
S	-0.1732640	-1.0542100	1.9314690
H	-4.5060330	-2.6948340	0.8728530
H	-4.1941870	-1.2494780	1.8424480
H	-3.7769940	1.6606080	-1.7580870
H	-2.9909680	0.5096560	-2.8187180
O	-0.5064720	0.9232230	-1.8928850
H	-1.6370210	2.4515560	-2.6101490

Ga(III)-Photoxenobactin E, thione-form:

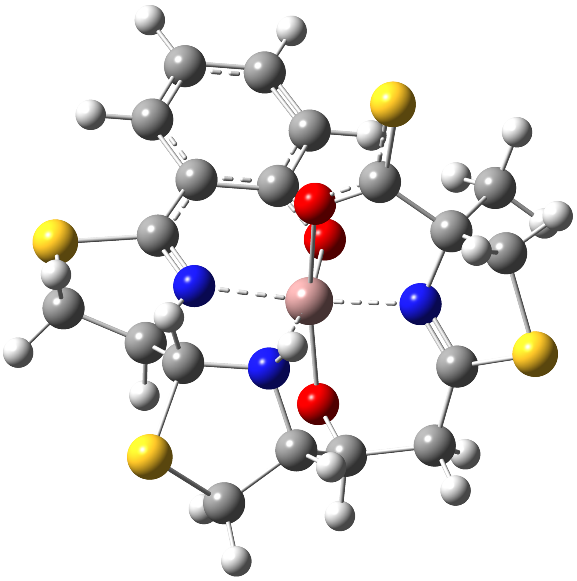

Figure S21. Ball and sticks model of Ga(III)-photoxenobactin E thione tautomer

Table S2. DFT data of the WB97XD/DEF2TVPP model Ga(III)-photoxenobactin E thione tautomer.

Zero-point correction= 0.381420 (Hartree/Particle)
Thermal correction to Energy= 0.407398
Thermal correction to Enthalpy= 0.408342
Thermal correction to Gibbs Free Energy= 0.326227
Sum of electronic and zero-point Energies= -4643.592388
Sum of electronic and thermal Energies= -4643.566410
Sum of electronic and thermal Enthalpies= -4643.565466
Sum of electronic and thermal Free Energies= -4643.647581

Number of Imaginary Frequencies = 0

Coordinates:

Symbol	X	Y	Z
C	3.0705770	-0.9720850	-0.3364570
C	2.1176000	-1.7784080	-1.0250530
C	2.5335930	-3.0648180	-1.4281400
C	3.7951180	-3.5357200	-1.1589810
C	4.7238070	-2.7465530	-0.4735440
C	4.3533130	-1.4861620	-0.0781820
C	2.7581050	0.3811260	0.0704600
S	3.9955780	1.5102680	0.5998240
C	2.7207420	2.6875270	1.1370130
C	1.4743700	2.2961810	0.3455900
N	1.5743390	0.8815280	0.0449440
O	0.9025350	-1.4106040	-1.3303770
H	1.8132360	-3.6753140	-1.9560840
H	4.0692050	-4.5327950	-1.4795140
H	5.7138210	-3.1209730	-0.2567650
H	5.0638320	-0.8710640	0.4595970
H	3.0487730	3.6989250	0.9205510
H	2.5745510	2.5755210	2.2102200
H	1.4574870	2.8265940	-0.6104240
Ga	-0.1083140	-0.0168320	-0.5147260
C	0.1563020	2.5619930	1.0932320
S	-0.3572280	4.2958740	0.9483710
C	-1.1183700	3.8865470	-0.6391850
C	-1.7459470	2.5009300	-0.4691150
N	-0.9418170	1.7552090	0.5366000
H	0.2800590	2.3056320	2.1432890
H	-0.3659390	3.8782380	-1.4272240
H	-1.8677850	4.6344010	-0.8827280
H	-2.7564140	2.6164580	-0.0793400
H	-1.5396240	1.4714090	1.2997990

C	-1.7629660	1.7192380	-1.8153300
C	-2.9878320	0.7821870	-1.9426510
C	-3.0148610	-0.3337220	-0.9507300
S	-4.5089450	-0.9647190	-0.3328720
C	-3.6105910	-1.9137680	0.9333650
C	-2.1516810	-1.9830040	0.4400020
N	-1.9536980	-0.8764670	-0.4913940
C	-1.8619900	-3.2912440	-0.3027010
H	-2.5685950	-3.4076690	-1.1252010
H	-1.9659240	-4.1329230	0.3788610
H	-0.8508940	-3.2751730	-0.7078080
C	-1.1123160	-1.8004800	1.5683110
O	-0.1731720	-1.0030070	1.3160990
S	-1.2737940	-2.6700580	2.9898520
H	-4.0511440	-2.9007240	1.0297000
H	-3.6998780	-1.3893330	1.8809270
H	-3.9127320	1.3544940	-1.8764970
H	-2.9587070	0.3311720	-2.9371650
O	-0.5655150	1.0567160	-2.0037730
H	-1.8997740	2.4677530	-2.6045310

References

[1] M. L. Lemos, P. Salinas, A. E. Toranzo, J. L. Barja, J. H. Crosa. Chromosome-mediated iron uptake system in pathogenic strains of *Vibrio anguillarum*, *J. Bacteriol.* **1988**, *170*, 1920–1925.

[2] A. Souto, M. A. Montaos, A. J. Rivas, M. Balado, C. R. Osorio, J. Rodríguez, M. L. Lemos, C. Jiménez. Structure and biosynthetic assembly of piscibactin, a siderophore from *Photobacterium damselae* subsp. *piscicida*, predicted from genome analysis. *European J. Org. Chem.* **2012**, *2012*, 5693–5700.

[3] Reich, H. J. WinDNMR: Dynamic NMR Spectra for Windows. *J. Chem. Educ.* **1995**, 72 (12), 1086.

[4] G. K. Pierens. ^{1}H and ^{13}C NMR scaling factors for the calculation of chemical shifts in commonly used solvents using density functional theory; *J. Comput. Chem.* **2014**, *35*, 1388–1394.
